# Serum human epididymis Protein-4 outperforms conventional biomarkers in the early detection of non-small cell lung cancer

**DOI:** 10.1016/j.isci.2024.111211

**Published:** 2024-10-19

**Authors:** Mohammad Erfan Zare, Atefeh Nasir Kansestani, Xuanlan Wu, Lin Zhou, Jie Lu, Jun Huang, Yanzhong Wang, Yilei Ma, Yuzhen Gao, Jun Zhang

**Affiliations:** 1 Department of Clinical Laboratory, Sir Run Run Shaw Hospital, Zhejiang University School of Medicine, Hangzhou, Zhejiang, P.R. China; 2 Key Laboratory of Precision Medicine in Diagnosis and Monitoring Research of Zhejiang Province, Hangzhou, Zhejiang, P.R. China

**Keywords:** Pathophysiology, Cancer

## Abstract

We employed a three-step approach to evaluate serum immunoassay-based biomarkers for detecting non-small cell lung cancer (NSCLC). In the first step, we performed a systematic review and meta-analysis and implemented the Laboratory Medicine Best Practices (LMBP) method to identify potential biomarkers. From potential biomarkers, Carcinoembryonic antigen (CEA), cytokeratin 19-fragments (Cyfra 21-1), and human epididymis protein-4 (HE4) were categorized as LMBP "recommend." In the second step, we conducted matched-case-control validation on these recommended biomarkers and SAA, identified as the most accurate in the first step. In the third step, a re-meta-analysis was performed by integrating our experimental results and considering covariates. The final results revealed that HE4 emerged as the most reliable biomarker, offering balanced sensitivity and specificity, with accuracy unaffected by tumor stage, making it suitable for early diagnosis. Our findings support the inclusion of HE4 in clinical guidelines for NSCLC diagnosis, alongside well-established biomarkers such as Cyfra 21-1 and CEA.

## Highlights

• HE4 emerged as the most reliable biomarker for the early detection of NSCLC
• HE4 accuracy for NSCLC was unaffected by tumor stage, unlike other markers
• Combining biomarkers did not improve accuracy over single markers in NSCLC

## Graphical abstract

**Figure:**
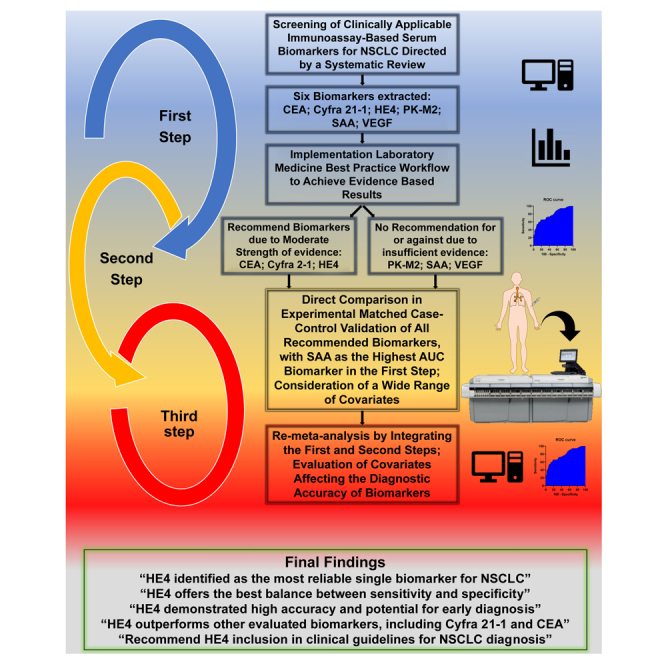

## Introduction

Lung cancer (LC) is one of the most prevalent malignancies worldwide and remains a leading cause of cancer-related mortality across both genders. Non-small-cell lung cancer (NSCLC) accounts for over 85% of all lung cancer cases and includes three primary histological subtypes: lung squamous cell carcinoma (LUSC), lung adenocarcinoma (LUAD), and lung large cell carcinoma (LLCC). The remaining cases consist of small cell lung cancer (SCLC) and other histological variants, each characterized by distinct biological behaviors and genetic mutations. Early-stage NSCLC can often be successfully treated with surgical resection, resulting in favorable outcomes.^1^^,^^2^ However, most lung cancers are diagnosed at advanced stages, leading to a poor 5-year survival rate, typically ranging between 10 and 20% globally. Therefore, early diagnosis is crucial for implementing effective treatment strategies for LC.^2^

Currently, the gold standard for diagnosing LC is biopsy, guided by thoracoscopy, bronchoscopy, or computed tomography (CT) scans. Despite their effectiveness, these methods are invasive, costly, and heavily dependent on the operator’s expertise. Consequently, there is a pressing need for the development of non-invasive, cost-effective diagnostic tools, such as serum biomarkers detected through immunoassay techniques.^1^^,^^3^ Several immunoassay-based serum biomarkers, including cytokeratin 19-fragments (Cyfra 21-1),^4^ human epididymis protein 4 (HE4),^5^^,^^6^ and carcinoembryonic antigen (CEA),^7^ have been proposed for the diagnosis of LC. However, the majority of these findings are derived from individual studies, which are often limited by small sample sizes and study design constraints.

Conducting a systematic review and meta-analysis of diagnostic test accuracy (DTA) by summarizing evidence from multiple studies provides a more precise estimate of test performance than what can be achieved through a single study.^8^ Additionally, implementing a comparative DTA method aid in identifying the most accurate and practical biomarker among the available options. Nevertheless, this approach has also some limitations, particularly when a biomarker is supported by only a small number of studies, or when the quality of the included studies is low, resulting in the exclusion of such biomarkers. Furthermore, the absence of sufficient data may lead to the omission of crucial covariates that could have an impact on the results.^9^ Also, indirect comparisons in DTA meta-analysis studies, while valuable, can be affected by confounding factors due to differences in patient groups and study methods, necessitating direct comparisons on the same samples to validate and enhance the reliability of the evaluations.^10^

The aim of this study was to identify the most accurate and practical immunoassay-based serum biomarkers for the diagnosis of NSCLC through a systematic, step-by-step approach. To address the aforementioned limitations and achieve the study’s objectives, we designed a comprehensive, three-step study. In the first step, we carried out an evidence-based comparative DTA meta-analysis to assess and compare the diagnostic accuracy of clinically applicable immunoassay-based serum biomarkers for the diagnosis of NSCLC using an indirect comparison approach. To achieve this objective, we employed the methodology recommended by the Laboratory Medicine Best Practices (LMBP) initiative of the Centers for Disease Control and Prevention (CDC) to translate systematic review findings into evidence-based recommendations.^11^^,^^12^ In the second step, to overcome the limitations of indirect comparisons in the first step, we conducted a matched case-control validation study to directly compare the accuracy of the biomarkers. This step was crucial to determine whether the comparison pattern of diagnostic accuracy observed in the indirect meta-analysis could be confirmed through direct comparison within the same sample set. In the last step, we integrated the results from the first and second steps to re-conduct the meta-analysis, considering a wide range of covariates to identify potential confounders.

## Results

### Step 1: The systematic review and meta-analysis results indicated that human epididymis protein-4 had the highest accuracy among the biomarkers classified as “recommend” by the laboratory medicine best practices method

**Figure 1:**
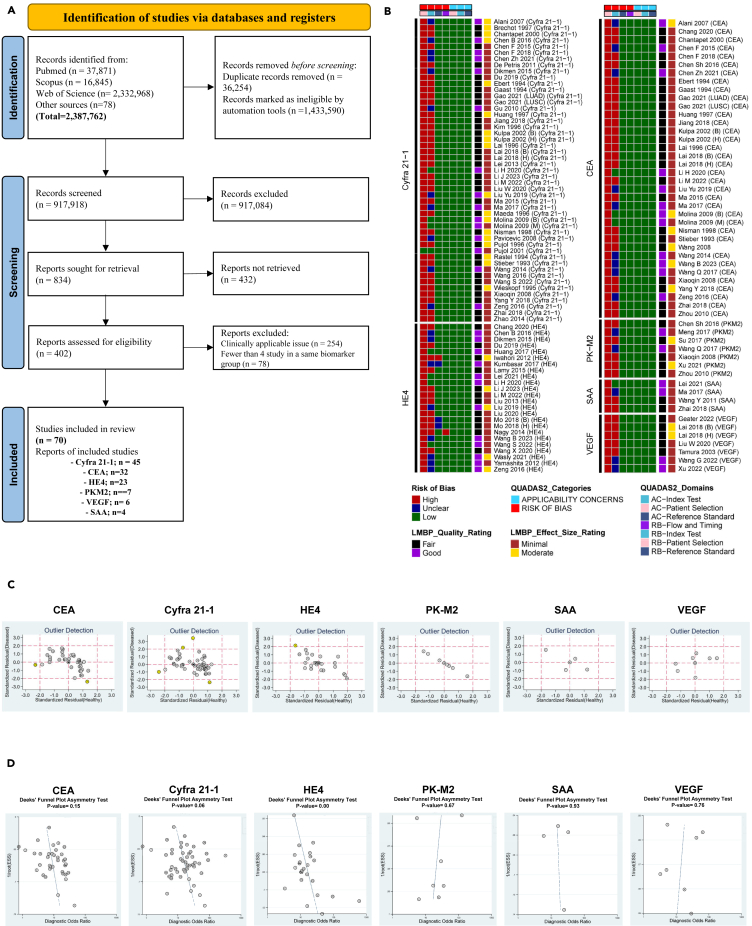
Overview and assessment of included studies (A) PRISMA flowchart illustrating the study selection process, detailing the number of records identified, screened, excluded, and included in the systematic review. (B) Heatmap of the quality assessment of included biomarkers using the QUADAS-2 tool and the final LMBP rating. Studies that received both a "good" LMBP quality rating (violet) and a "moderate" LMBP effect size rating (yellow) were considered for further steps in the LMBP workflow. (C) Evaluation of outliers among included biomarkers, with outlier studies highlighted in yellow. (D) Publication bias analysis represented by Deeks' Funnel Plot Asymmetry Tests for each biomarker. A *p*-value of less than 0.1 indicates significant publication bias. **Abbreviations:** QUADAS-2, Quality Assessment of Diagnostic Accuracy Studies 2; LMBP, Laboratory Medicine Best Practice; CEA, Carcinoembryonic Antigen; HE4, Human Epididymis Protein 4; SAA, Serum Amyloid A; PK-M2, Pyruvate Kinase M2; VEGF, Vascular Endothelial Growth Factor.

Seventy studies, encompassing 51,563 test results, were selected from an initial 2,387,762 records based on exclusion criteria (Figure 1A). After removing 36,254 studies due to duplication and excluding 1,433,590 studies using filters for article type (e.g., review articles, editorials, case reports, clinical trials, and clinical guidelines) and species (human), 917,918 records underwent title and abstract screening. At this stage, 917,084 studies were excluded. The primary reasons for exclusion were (1) biomarkers evaluated on non-serum samples, such as bronchoalveolar lavage or tissue, and (2) non-immunoassay-based techniques, including molecular biomarkers. Out of the remaining 402 reports, 332 studies were excluded because the biomarkers did not meet the criteria for clinical applicability (e.g., progastrin-releasing peptide [ProGRP], neuron-specific enolase [NSE], squamous cell carcinoma antigen [SCCA], cancer antigen 125 [CA 125], serum P53) or had fewer than four studies within the same biomarker group (e.g., complement activation product C4d [C4d], matrix metallopeptidase 9 [MMP-9]). Ultimately, the final analysis included 70 studies. According to the inclusion and exclusion criteria, CEA,^13^^,^^14^^,^^15^^,^^16^^,^^17^^,^^18^^,^^19^^,^^20^^,^^21^^,^^22^^,^^23^^,^^24^^,^^25^^,^^26^^,^^27^^,^^28^^,^^29^^,^^30^^,^^31^^,^^32^^,^^33^^,^^34^^,^^35^^,^^36^^,^^37^^,^^38^^,^^39^^,^^40^^,^^41^^,^^42^^,^^43^^,^^44^ Cyfra 21-1^13^^,^^15^^,^^16^^,^^17^^,^^19^^,^^20^^,^^21^^,^^22^^,^^23^^,^^24^^,^^25^^,^^26^^,^^27^^,^^28^^,^^29^^,^^30^^,^^31^^,^^32^^,^^33^^,^^34^^,^^35^^,^^37^^,^^40^^,^^41^^,^^42^^,^^43^^,^^45^^,^^46^^,^^47^^,^^48^^,^^49^^,^^50^^,^^51^^,^^52^^,^^53^^,^^54^^,^^55^^,^^56^^,^^57^^,^^58^^,^^59^^,^^60^^,^^61^^,^^62^^,^^63^ HE4,^14^^,^^28^^,^^29^^,^^30^^,^^38^^,^^42^^,^^46^^,^^48^^,^^49^^,^^53^^,^^54^^,^^64^^,^^65^^,^^66^^,^^67^^,^^68^^,^^69^^,^^70^^,^^71^^,^^72^^,^^73^^,^^74^ pyruvate kinase-M2 (PK-M2),^18^^,^^39^^,^^40^^,^^44^^,^^75^^,^^76^^,^^77^ serum amyloid A (SAA),^32^^,^^43^^,^^68^^,^^78^ and vascular endothelial growth factor (VEGF)^27^^,^^54^^,^^79^^,^^80^^,^^81^^,^^82^ were identified as eligible biomarkers for further assessment. Additionally, four studies included healthy and benign lung disease control groups, assessing the biomarkers' accuracy separately for these groups. Therefore, two separate 2 × 2 contingency tables were constructed based on these papers.^25^^,^^27^^,^^33^^,^^70^ Table 1 provides a summary of the main characteristics of the included studies in this step.

**Table 1:** Characteristics of included studies

Study ID	Language	Country	Design	Study population		Method	Cut-off	Control group				% Smoker in Patients	% Smokers in control	%Late	Histologic type		
				Age	%Male			%H	%B	%SCLC	%M				% LUAD	% LLCC	% LUSC
**CEA**																	

Alani 2007	Persian	Iran	CC	65	NA	ELISA	5	0	100	0	0	NA	NA	NA	22.3	27.7	50
Chang 2020	Chinese	China	CC	58.5	57.8	CLIA	5.82	100	0	0	0	NA	NA	0	55.2	0	44.8
Chantapet 2000	English	Thailand	CC	60.5	62.3	ELISA	7.7	0	100	0	0	NA	NA	67.2	67.2	1.8	23.6
Chen F 2015	English	China	CC	56.5	51.9	CLIA	2.13	0	100	0	0	73.4	63.3	9.4	74.1	0	23.7
Chen F 2018	English	China	CC	57.5	57.1	CLIA	5	0	100	0	0	NA	NA	25.4	72.9	0	27.1
Chen Sh 2016	Chinese	China	CC	NA	NA	CLIA	5	100	0	0	0	NA	NA	63.5	55.2	0	44.8
Chen Zh 2021	English	China	CC	62.5	56.5	CLIA	3.2	0	0	100	0	42.9	70.3	28.1	70.1	NA	19.7
Ebert 1994	English	Germany	CC	57.5	60.8	ELISA	7.8	0	100	0	0	76.8	19.9	69.3	35.5	NA	45.7
Gaast 1994	English	Belgium	CC	NA	NA	ELISA	7.4	0	100	0	0	NA	NA	NA	30.7	31.6	37.7
Gao 2021 (LUAD)	Chinese	China	CC	64.9	60.7	CLIA	5.34	0	100	0	0	40.1	34.3	NA	100	0	0
Gao 2021 (LUSC)	Chinese	China	CC	66.2	80.4	CLIA	3.82	0	100	0	0	74.4	34.4	NA	0	0	100
Huang 1997	English	Taiwan	CC	NA	NA	RIA	5	0	100	0	0	NA	NA	79.4	51.7	7	41.3
Jiang 2018	English	China	CC	NA	NA	CLIA	4.94	0	100	0	0	NA	NA	NA	0	0	100
Kulpa 2002 (B)	English	Poland	CC	NA	NA	CLIA	4.1	0	100	0	0	NA	NA	75.5	0	0	100
Kulpa 2002 (H)	English	Poland	CC	NA	NA	CLIA	4.1	100	0	0	0	NA	NA	75.5	0	0	100
Lai 1996	English	China	CC	NA	NA	ELISA	7.8	0	100	0	0	NA	NA	NA	38.8	8	53.2
Lai 2018 (B)	English	China	CC	NA	56.6	ELISA	4.65	0	100	0	0	NA	NA	36.6	NA	NA	NA
Lai 2018 (H)	English	China	CC	NA	53.8	ELISA	3.37	100	0	0	0	NA	NA	36.6	NA	NA	NA
Li H 2020	Chinese	China	CC	51	54.7	CLIA	2.3	100	0	0	0	NA	NA	20.5	74	0	26
Li M 2022	English	China	CC	NA	NA	CLIA	3.48	76.7	13.2	0	2.9	NA	NA	NA	63.6	6.5	29.9
Liu Yu 2019	Chinese	China	CC	NA	NA	Chip	NA	25.5	26.5	29	19	NA	NA	NA	NA	NA	NA
Ma 2015	English	China	CC	54.5	44.6	CLIA	3.4	50	50	0	0	NA	NA	60.7	42.8	0	57.1
Ma 2017	English	China	CC	NA	NA	CLIA	NA	100	0	0	0	NA	NA	NA	NA	NA	NA
Molina 2009 (B)	English	Spain	CC	NA	NA	ELISA	5	0	100	0	0	NA	NA	NA	43.4	4	38.5
Molina 2009 (M)	English	Spain	CC	NA	NA	ELISA	5	0	47	53	0	NA	NA	NA	43.4	4	38.5
Nisman 1998	English	Israel	CC	60	60.3	RIA	4.7	0	100	0	0	NA	NA	75.5	43.6	13.9	42.5
Stieber 1993	English	Germany	CC	NA	NA	ELISA	7.4	NA	NA	NA	NA	NA	NA	NA	NA	NA	NA
Wang 2008	Chinese	China	CC	NA	NA	CLIA	3	54.4	45.6	0	0	NA	NA	88.2	63.2	9.5	22.7
Wang 2014	English	China	CC	NA	NA	CLIA	9.8	63.3	36.7	0	0	NA	NA	NA	48.4	0	51.6
Wang B 2023	Chinese	China	CC	NA	NA	CLIA	NA	100	0	0	0	NA	AN	50.5	82.4	NA	17.5
Wang Q 2017	Chinese	China	CC	NA	NA	CLIA	5	100	0	0	0	NA	NA	60	53.4	0	46.6
Xiaoqin 2008	Chinese	China	CC	NA	NA	ELISA	4.1	0	100	0	0	NA	NA	71.5	55.7	0	44.3
Yang Y 2018	English	China	CC	56.04	52.5	CLIA	4.21	0	100	0	0	55.7	42	54.8	56.7	NA	38.4
Zeng 2016	English	China	CC	NA	63.3	CLIA	5.82	100	0	0	0	NA	NA	0	56.7	0	43.3
Zhai 2018	Chinese	China	CC	NA	NA	CLIA	4.9	0	100	0	0	48	NA	29.4	NA	NA	NA
Zhou 2010	Chinese	China	CC	NA	NA	CLIA	5	50	50	0	0	NA	NA	66.6	57	0	43

**Cyfra 21-1**																	

Alani 2007	Persian	Iran	CC	65	NA	ELISA	3.5	0	100	0	0	NA	NA	NA	22.3	27.7	50
Bréchot 1997	English	France	CC	NA	NA	ELISA	3.3	0	100	0	0	NA	NA	84.4	43.9	12.9	42.2
Chantapet 2000	English	Thailand	CC	60.5	62.3	ELISA	3.13	0	100	0	0	NA	NA	67.2	67.2	1.8	23.6
Chen B 2016	Chinese	China	CC	NA	NA	CLIA	NA	52.7	47.3	0	0	NA	NA	62	NA	NA	NA
Chen F 2015	English	China	CC	56.5	51.9	CLIA	2.54	0	100	0	0	73.4	63.3	9.4	74.1	0	23.7
Chen F 2018	English	China	CC	57.5	57.1	CLIA	3.3	0	100	0	0	NA	NA	25.4	72.9	0	27.1
Chen Zh 2021	English	China	CC	62.5	56.5	CLIA	3	0	0	100	0	42.9	70.3	28.1	70.1	NA	19.7
De Petris 2011	English	Sweden	CC	NA	NA	ELISA	3.6	0	100	0	0	90	NA	62	NA	NA	29
Dikmen 2015	English	Turky	CC	56.6	73.7	CLIA	2	0	100	0	0	NA	NA	47.2	49	0	51
Du 2019	Chinese	China	CC	NA	NA	ELISA	19.13	100	0	0	0	NA	NA	62	NA	NA	NA
Ebert 1994	English	Germany	CC	57.5	60.8	ELISA	3.3	0	100	0	0	76.8	19.9	69.3	35.5	NA	45.7
Gaast 1994	English	Belgium	CC	NA	NA	ELISA	3.3	0	100	0	0	NA	NA	NA	30.7	31.6	37.7
Gao 2021 (LUAD)	Chinese	China	CC	64.9	60.7	CLIA	3.36	0	100	0	0	40.1	34.3	NA	100	0	0
Gao 2021 (LUSC)	Chinese	China	CC	66.2	80.4	CLIA	4.63	0	100	0	0	74.4	34.4	NA	0	0	100
Gu 2010	Chinese	China	CC	54.5	59.4	ELISA	3.3	0	100	0	0	NA	NA	42.1	47.7	NA	44.9
Huang 1997	English	Taiwan	CC	NA	NA	RIA	3.5	0	100	0	0	NA	NA	79.4	51.7	7	41.3
Jiang 2018	English	China	CC	NA	NA	CLIA	3.71	0	100	0	0	NA	NA	NA	0	0	100
Kim 1996	English	Korea	CC	NA	NA	RIA	3.3	0	100	0	0	NA	NA	NA	NA	NA	NA
Kulpa 2002 (B)	English	Poland	CC	NA	NA	CLIA	3.92	0	100	0	0	NA	NA	75.5	0	0	100
Kulpa 2002 (H)	English	Poland	CC	NA	NA	CLIA	2.48	100	0	0	0	NA	NA	75.5	0	0	100
Lai 1996	English	China	CC	NA	NA	ELISA	3.3	0	100	0	0	NA	NA	NA	38.8	8	53.2
Lai 2018 (B)	English	China	CC	NA	56.6	ELISA	2.82	0	100	0	0	NA	NA	36.6	NA	NA	NA
Lai 2018 (H)	English	China	CC	NA	53.8	ELISA	3.34	100	0	0	0	NA	NA	36.6	NA	NA	NA
Liu 2013	English	China	CC	52	60	CLIA	3.3	0	100	0	0	NA	NA	NA	0	0	100
Li H 2020	Chinese	China	CC	51	54.7	CLIA	2.17	100	0	0	0	NA	NA	20.5	74	0	26
Li J 2023	English	China	CC	57.7	48.4	CLIA	3.14	100	0	0	0	NA	NA	NA	86.1	0	13.9
Li M 2022	English	China	CC	NA	NA	CLIA	2.77	76.7	13.2	0	2.9	NA	NA	NA	63.6	6.5	29.9
Liu W 2020	Chinese	China	CC	51	54.7	ELISA	9.6	50	50	0	0	NA	NA	55	53.4	0	46.6
Liu Yu 2019	Chinese	China	CC	NA	NA	chip	NA	25.5	26.5	29	19	NA	NA	NA	NA	NA	NA
Ma 2015	English	China	CC	54.5	44.6	CLIA	3.3	50	50	0	0	NA	NA	60.7	42.8	0	57.1
Ma 2017	English	China	CC	NA	NA	CLIA	NA	100	0	0	0	NA	NA	NA	NA	NA	NA
Maeda 1996	English	Japan	CC	NA	NA	ELISA	3.5	0	100	0	0	NA	NA	67.43	44.9	4.5	50.45
Molina 2009 (B)	English	Spain	CC	NA	NA	ELISA	3.3	0	100	0	0	NA	NA	NA	43.4	4	38.5
Molina 2009 (M)	English	Spain	CC	NA	NA	ELISA	3.3	0	47	53	0	NA	NA	NA	43.4	4	38.5
Nisman 1998	English	Israel	CC	60	60.3	RIA	3.2	0	100	0	0	NA	NA	75.5	43.6	13.9	42.5
Pavicevic 2008	English	Croatia	CC	NA	NA	CLIA	1.72	0	100	0	0	NA	NA	31	37.9	8.2	51
Pujol 1996	English	France	CC	NA	NA	RIA	3.6	0	100	0	0	NA	NA	91	24.2	13.05	62.73
Pujol 2001	English	France	C	NA	NA	RIA	3.6	0	100	0	0	NA	NA	NA	26.8	14	58.2
Rastel 1994	English	Belgium	CC	58	NA	RIA	3.3	0	100	0	0	NA	NA	NA	31.4	18	50.6
Stieber 1993	English	Germany	CC	NA	NA	ELISA	2.1	NA	NA	NA	NA	NA	NA	NA	NA	NA	NA
Wang 2014	English	China	CC	NA	NA	CLIA	3.5	63.3	36.7	0	0	NA	NA	NA	48.4	0	51.6
Wang 2016	English	China	CC	56.75	69.4	CLIA	6.59	0	100	0	0	NA	NA	40.6	44	5	50.8
Wang S 2022	Chinese	China	CC	NA	NA	CLIA	6.43	100	0	0	0	NA	NA	61.7	59	NA	41
Wieskopf 1995	English	France	CC	NA	NA	RIA	3.3	0	100	0	0	NA	NA	88	NA	NA	62
Xiaoqin 2008	Chinese	China	CC	NA	NA	ELISA	3.8	0	100	0	0	NA	NA	71.5	55.7	0	54.3
Yang Y 2018	English	China	CC	56.04	52.5	CLIA	2.7	0	100	0	0	55.7	42	54.8	56.7	NA	38.4
Zeng 2016	English	China	CC	NA	63.3	CLIA	3.79	100	0	0	0	NA	NA	0	56.7	0	43.3
Zhai 2018	Chinese	China	CC	NA	NA	CLIA	9.7	0	100	0	0	48	NA	29.4	NA	NA	NA
Zhao 2014	English	China	CC	NA	NA	ELISA	6.32	0	100	0	0	NA	NA	80.3	50	NA	45.4

**HE4**																	

Chang 2020	Chinese	China	CC	58.5	57.8	CLIA	23.82	100	0	0	0	NA	NA	0	55.2	0	44.8
Chen B 2016	Chinese	China	CC	NA	NA	CLIA	NA	52.7	47.3	0	0	NA	NA	62	NA	NA	NA
Dikmen 2015	English	Turkey	CC	56.6	73.7	CLIA	70	0	100	0	NA	NA	NA	47.2	49	0	51
Du 2019	Chinese	China	CC	NA	NA	ELISA	288.32	100	0	0	0	NA	NA	62	NA	NA	NA
Huang 2017	English	China	CC	NA	NA	CLIA	75	100	0	0	NA	NA	NA	NA	NA	NA	NA
Iwahori 2012	English	Japan	CC	NA	NA	ELISA	6.56	100	0	0	NA	NA	NA	NA	82.5	2.5	15
Kumbasar 2017	English	Turkey	CC	NA	NA	CLIA	70	0	100	0	NA	NA	NA	NA	NA	NA	NA
Lamy 2015	English	France	CC	61	NA	ELISA	53	0	100	0	NA	NA	NA	91.3	27.2	15	57.8
Lei 2021	Chinese	China	CC	NA	NA	CLIA	80	50.5	49.5	0	0	NA	NA	67	45.2	5.6	46.2
Li H 2020	Chinese	China	CC	51	54.7	CLIA	45.5	100	0	0	0	NA	NA	20.5	74	0	26
Li J 2023	English	China	CC	57.7	48.5	CLIA	60.15	100	0	0	NA	NA	NA	NA	86.1	0	13.9
Li M 2022	English	China	CC	NA	NA	CLIA	60.37	76.7	13.2	0	2.9	NA	NA	NA	63.6	6.5	29.6
Liu 2013	English	China	CC	58.2	74.5	ELISA	83.9	43.4	56.6	0	NA	55	55.3	69.8	NA	NA	NA
Liu 2019	Chinese	China	CC	NA	NA	ELISA	NA	25.5	26.5	29	19	NA	NA	NA	NA	NA	NA
Liu 2020	Chinese	China	CC	51	54.7	ELISA	105.89	50	50	0	NA	NA	NA	55	53.4	0	46.6
Mo 2018 (B)	English	China	CC	NA	NA	CLIA	81.26	0	100	0	NA	NA	NA	NA	NA	NA	NA
Mo 2018 (H)	English	China	CC	NA	NA	CLIA	78.84	100	0	0	NA	NA	NA	NA	NA	NA	NA
Nagy 2014	English	Hungary	CC	NA	100	CLIA	79.65	100	0	0	NA	NA	NA	NA	44.9	7.3	47.8
Wang B 2023	Chinese	China	CC	55.9	63.8	ELISA	NA	100	0	0	0	NA	NA	50.5	80.4	NA	17.5
Wang S 2022	Chinese	China	CC	NA	NA	CLIA	73.12	100	0	0	0	NA	NA	61.7	59	0	41
Wang X 2020	Chinese	China	CC	65	62.8	CLIA	195.36	100	0	0	0	52	NA	65.5	56.3	0	43.7
Wasly 2021	English	Egypt	CC	NA	NA	ELISA	60	100	0	0	NA	NA	NA	NA	NA	NA	NA
Yamashita 2012	English	Japan	CC	NA	NA	ELISA	50.3	78.3	21.7	0	NA	NA	NA	NA	100	0	0
Zeng 2016	English	China	CC	NA	63.3	CLIA	66.8	100	0	0	NA	NA	NA	0	56.7	0	43.3

**PK-M2**																	

Chen Sh 2016	Chinese	China	CC	NA	NA	ELISA	NA	100	0	0	0	NA	NA	63.5	55.2	0	44.8
Meng 2017	Chinese	China	CC	63.5	59.7	ELISA	NA	0	100	0	0	NA	NA	NA	NA	NA	NA
Su 2017	Chinese	China	CC	NA	NA	ELISA	15.78	100	0	0	0	NA	NA	NA	NA	NA	NA
Wang Q 2017	Chinese	China	CC	NA	NA	ELISA	15	100	0	0	0	NA	NA	60	53.4	0	46.6
Xiaoqin 2008	Chinese	China	CC	NA	NA	ELISA	13	0	100	0	0	NA	NA	71.5	55.7	0	44.3
Xu 2021	English	China	CC	55.8	46.2	ELISA	30	67.2	32.8	0	0	52	NA	65.5	77.8	0	22.2
Zhou 2010	Chinese	China	CC	NA	NA	ELISA	16.5	50	50	0	0	NA	NA	66.6	57	0	43

**SAA**																	

Lei 2021	Chinese	China	CC	NA	NA	Agglutination	10	50.5	49.5	0	0	NA	NA	67	45.2	5.6	46.2
Ma 2017	English	China	CC	NA	NA	CLIA	NA	100	0	0	0	NA	NA	NA	NA	NA	NA
Wang Y 2011	Chinese	China	CC	55.6	NA	ELISA	NA	100	0	0	0	NA	NA	53.5	34.9	NA	48.9
Zhai 2018	Chinese	China	CC	NA	NA	ELISA	28.4	0	100	0	0	48	NA	29.4	NA	NA	NA

**VEGF**																	

Geater 2022	English	Thailand	CC	NA	NA	ELISA	NA	0	100	0	0	NA	NA	NA	NA	NA	NA
Lai 2018 (B)	English	China	CC	NA	56.6	ELISA	78.7	0	100	0	0	NA	NA	36.6	NA	NA	NA
Lai 2018 (H)	English	China	CC	NA	53.8	ELISA	79.3	100	0	0	0	NA	NA	36.6	NA	NA	NA
Liu W 2020	Chinese	China	CC	51	54.7	ELISA	72.18	50	50	0	0	NA	NA	55	53.4	0	46.6
Tamura 2003	English	Japan	CC	60.5	NA	ELISA	327.8	42	58	0	0	NA	NA	33.6	61.9	NA	31.5
Wang G 2022	Chinese	China	CC	NA	NA	ELISA	160	100	0	0	0	NA	NA	NA	NA	NA	NA
Xu 2022	English	China	CC	68.3	43.4	ELISA	465.6	100	0	0	0	NA	NA	75.5	69.9	0	30.1

CEA: Carcinoma embryonic antigen; Cyfra 21-1: Cytokeratin 19-fragments; HE4: Human epididymis protein-4; SAA: Serum amyloid-A; NSCLC: Non-small-cell lung cancer; LUAD: Lung adenocarcinoma; LUSC: Lung squamous cell carcinoma; LLCC: Lung large cell carcinoma; B: benign lung disease; H: Healthy controls; M; non-lung malignancies.

The Quality Assessment of Diagnostic Accuracy Studies-2 (QUADAS-2) evaluation for each biomarker is illustrated in Figure 1B. The assessment revealed a high risk of bias in the "patient selection" and "index test" categories across all six biomarker groups, primarily due to the use of case-control study designs and the lack of blinding to reference standard results. However, no concerns were raised regarding the applicability of the included studies for any of the biomarkers. Outlier detection analysis identified outlier studies for CEA, Cyfra 21-1, and HE4, but none for PK-M2, VEGF, or SAA (Figure 1C). These outlier studies were included in the subsequent outlier exclusion and sensitivity analyses. Specifically, the CEA group had two outlier studies, Cyfra 21-1 had four, and HE4 had one. The Deeks' funnel plot asymmetry test indicated no significant publication bias in the CEA, PK-M2, SAA, or VEGF datasets. However, significant publication bias was observed in the Cyfra 21-1 and HE4 datasets (Figure 1D).

**Figure 2:**
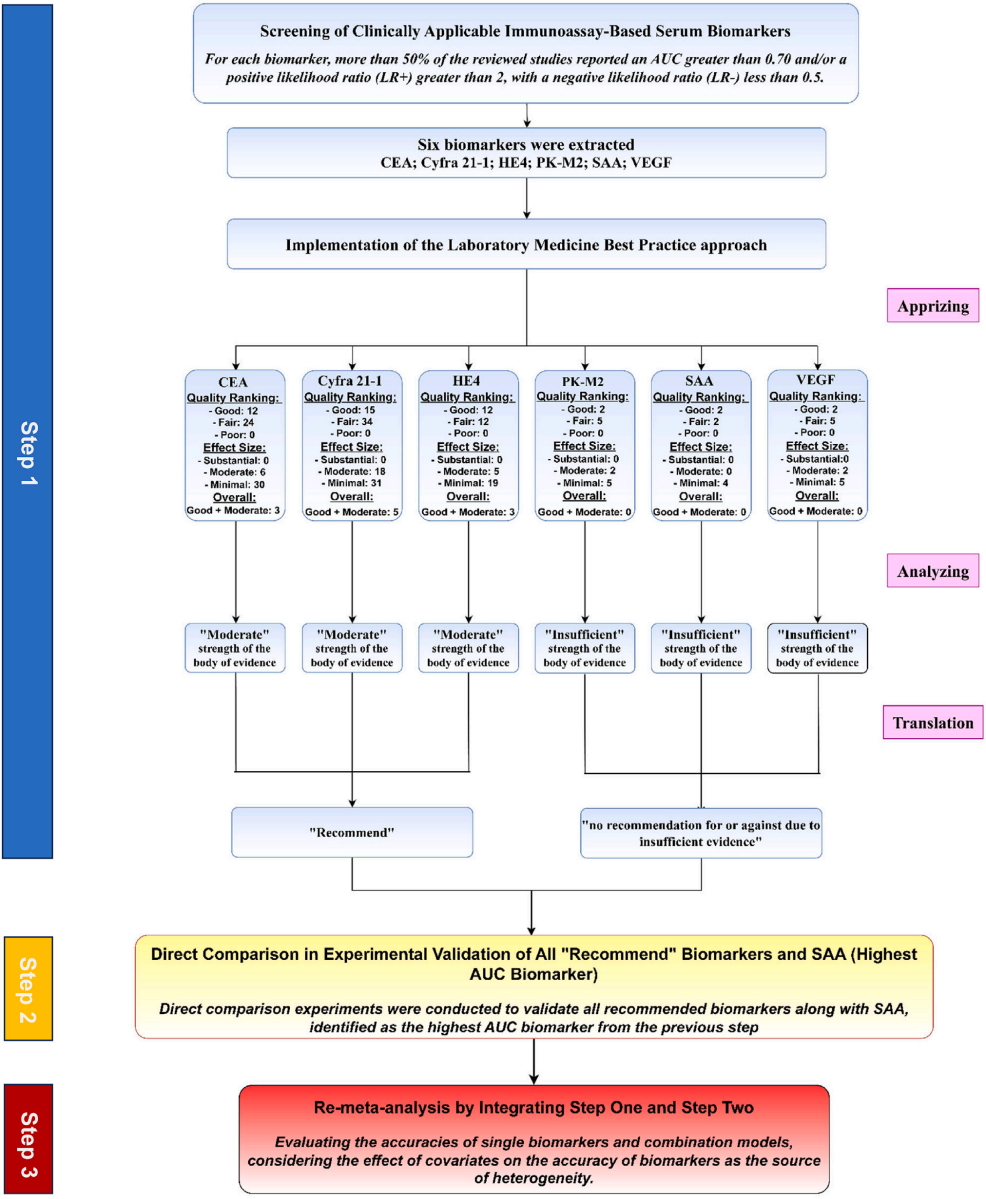
Flowchart outlining the sequential steps of the study This flowchart illustrates the step-by-step methodology employed throughout the study, including data collection, analysis, and validation phases **Abbreviations:** CEA, Carcinoembryonic Antigen; HE4, Human Epididymis Protein 4; SAA, Serum Amyloid A; PK-M2, Pyruvate Kinase M2; VEGF, Vascular Endothelial Growth Factor; LR, Likelihood Ratio; AUC, Area Under the Curve.

After identifying these six serum biomarkers, the subsequent step involved implementing the LMBP method. Based on the LMBP criteria, 3 studies for CEA, 5 for Cyfra 21-1, and 3 for HE4 received both a “good” LMBP quality rating and a “moderate” effect size rating (Figure 1B). None of the studies were ranked as “poor” quality, and none of the studies showed a “substantial” effect size, with the highest-ranked studies for all biomarkers being moderate. Then, the results of the Apprizing step were summarized to classify the overall strength of the evidence. Based on these results, in the Analyzing step, CEA, Cyfra 21-1, and HE4 were ranked as having “Moderate” strength of evidence, while PK-M2, SAA, and VEGF were considered to have “Insufficient” evidence. According to the LMBP evidence-based recommendation categories, CEA, Cyfra 21-1, and HE4 were classified as “recommend,” while the other three biomarkers received “no recommendation for or against due to insufficient evidence.” Figure 2 depicts the diagram of the LMBP method implemented in our study.

**Figure 3:**
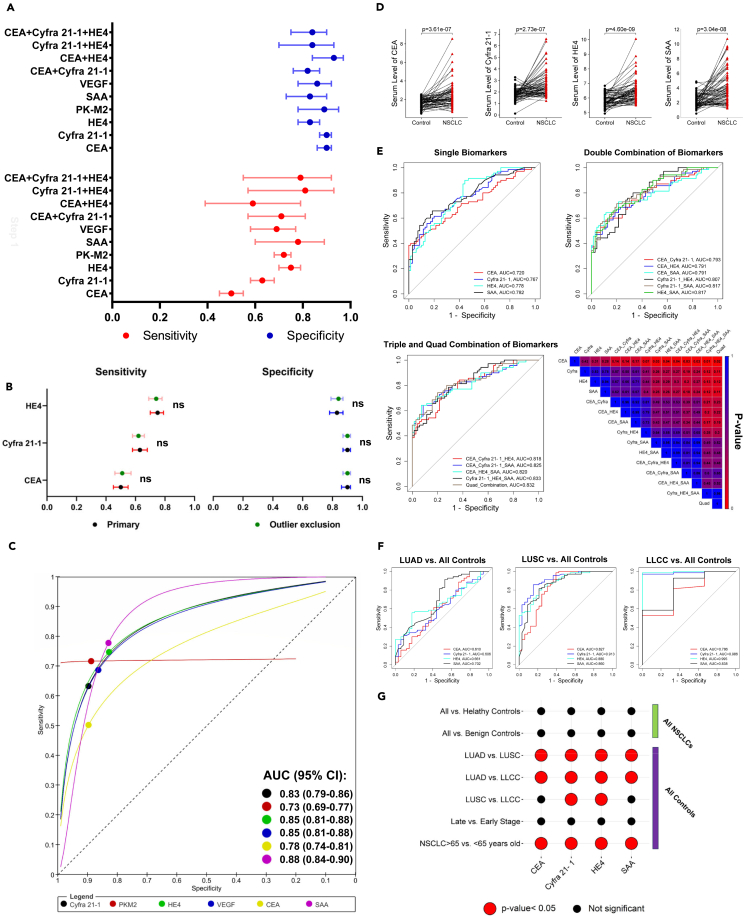
Analysis of Biomarker Performance and Diagnostic Accuracy (A) The pooled sensitivity and specificity of biomarkers and their combinations before outlier exclusion. (B) The effect of outlier exclusion on the pooled sensitivity and specificity of biomarkers. (C) ROC curve analysis of included biomarkers during the first step of the meta-analysis after outlier exclusion. (D) Log2-transformed serum levels of biomarkers in a case-control matched setup, based on data from the experimental validation step. Each line represents an NSCLC patient (*n* = 70) and its matched control (*n* = 70) sample. (E) ROC curves of single, double, triple, and quadruple biomarker combinations, along with a heatmap of biomarker comparison *p*-values, based on data from the experimental validation step. (F) ROC curves of NSCLC histologic subtypes for each single biomarker, based on data from the experimental validation step. (G) The effect of different covariates—including control sample composition, NSCLC histologic subtype, tumor stage, and patient age—on the diagnostic accuracy of biomarkers. Data in the forest plots are presented with 95% confidence intervals. The effect of outlier exclusion was compared using a standard bivariate comparison method. Serum biomarker comparisons between NSCLC and control group samples were conducted using a paired Student’s t test (or, when applicable, its non-parametric equivalent, the Wilcoxon test). Accuracy comparisons in the experimental validation step were performed using AUC comparison, as described by Hanley & McNeil. **Abbreviations:** LUAD, Lung Adenocarcinoma; LUSC, Lung Squamous Cell Carcinoma; LLCC, Large-Cell Lung Carcinoma; ROC, Receiver Operating Characteristic; NSCLC, Non-Small Cell Lung Cancer; CEA, Carcinoembryonic Antigen; HE4, Human Epididymis Protein 4; SAA, Serum Amyloid A; PK-M2, Pyruvate Kinase M2; VEGF, Vascular Endothelial Growth Factor; AUC, Area Under the Curve; ns: not significant.

The overall results of the meta-analysis from the first step are summarized in Figures 3A–3C. These results demonstrated that SAA has the highest Area Under the Curve (AUC) with a value of 0.88 (95% CI: 0.84–0.90) for the detection of NSCLC, followed by HE4, Cyfra 21-1, and VEGF (Figure 3C and Table S1). All biomarkers had significantly superior diagnostic performance compared to CEA (Figure 3C and Table S2).

A notable difference was observed in the diagnostic performance between Cyfra 21-1 and HE4. The sensitivity of HE4 was significantly higher than that of Cyfra 21-1 (relative sensitivity: 1.19 (95% CI: 1.07–1.32), *p*-value< 0.01), while the specificity of HE4 was significantly lower than that of Cyfra 21-1 (relative specificity: 0.93 (95% CI: 0.87–0.99, *p*-value = 0.02). This disparity resulted in a significant difference in the global test comparison between HE4 and Cyfra 21-1 for the diagnosis of NSCLC (*p*-value <0.01). The diagnostic performance of the other biomarkers did not show any significant differences compared with each other (Table S2).

To compare the diagnostic performance of the most accurate biomarker and the biomarkers recommended by LMBP in achieving more accurate results, we initially excluded any outlier studies and subsequently recalculated their diagnostic accuracies. The results showed that there were no significant differences in diagnostic performances after excluding outliers for biomarkers when compared to all included studies (Figure 3B and Table S3). After these modifications, among the LMBP recommended biomarkers, HE4 exhibited the highest AUC for diagnosing NSCLC, although still lower than SAA. SAA and HE4 demonstrated significantly higher overall accuracy compared to CEA (*p*-value <0.01 and <0.001, respectively), with only HE4 showing higher overall accuracy compared to Cyfra 21-1 (*p*-value <0.01). Furthermore, there were no significant differences between the sensitivity, specificity, and overall accuracy of HE4 and SAA (Table S2).

The combination of biomarkers did not improve the performance of NSCLC detection compared to SAA, which is the highest overall accurate biomarker, or HE4, which is the highest LMBP "recommend" biomarker, both before and after implementing outlier exclusion (Figure 3A). Details regarding the diagnostic accuracy of single biomarkers and their combinations can be found in Tables S1 and S2.

### Step 2: Experimental comparison validates meta-analysis pattern for biomarker accuracy in non-small cell lung cancer detection

Based on the findings of the preceding meta-analysis, four biomarkers—SAA, CEA, Cyfra 21-1, and HE4—underwent experimental direct comparison validation (Figure 2). Information on the characteristics of the study subjects can be found in Table S4.

All four evaluated biomarkers showed significant increases in NSCLC compared to matched control samples (Figure 3D). Consistent with the previous step, SAA demonstrated the highest diagnostic accuracy for detecting NSCLC, followed by HE4, Cyfra 21-1, and CEA (Figure 3E). However, there were no significant differences among the single biomarkers in terms of diagnostic accuracy (Figure 3E).

Despite slightly better performance with the combination of the aforementioned biomarkers, their accuracy was not significantly improved compared to SAA, HE4, and Cyfra 21-1 as single biomarkers. However, combinations of HE4+SAA and Cyfra 21-1+SAA, as well as all forms of triple and quadruple combinations, exhibited significantly higher accuracy compared to single CEA for NSCLC detection (Figure 3E).

Among the evaluated covariates, NSCLC histological subtypes significantly affected the accuracy of all biomarkers. All four biomarkers performed better in detecting LUSC and LLCC compared to LUAD (Figure 3F and 3G). Additionally, age significantly affected the accuracy of all biomarkers, with patients over the age of 65 showing higher detection accuracy across all single biomarkers compared to younger individuals (Figure 3G). The tumor stage and the composition of control samples did not significantly alter the diagnostic accuracy of the biomarkers (Figure 3G). Detailed results on the diagnostic accuracy of biomarkers and their comparisons are provided in Tables S5 and S6.

### Step 3: Final meta-analysis results reveal human epididymis protein-4 as the most accurate biomarker for the early detection of non-small cell lung cancer

**Figure 4:**
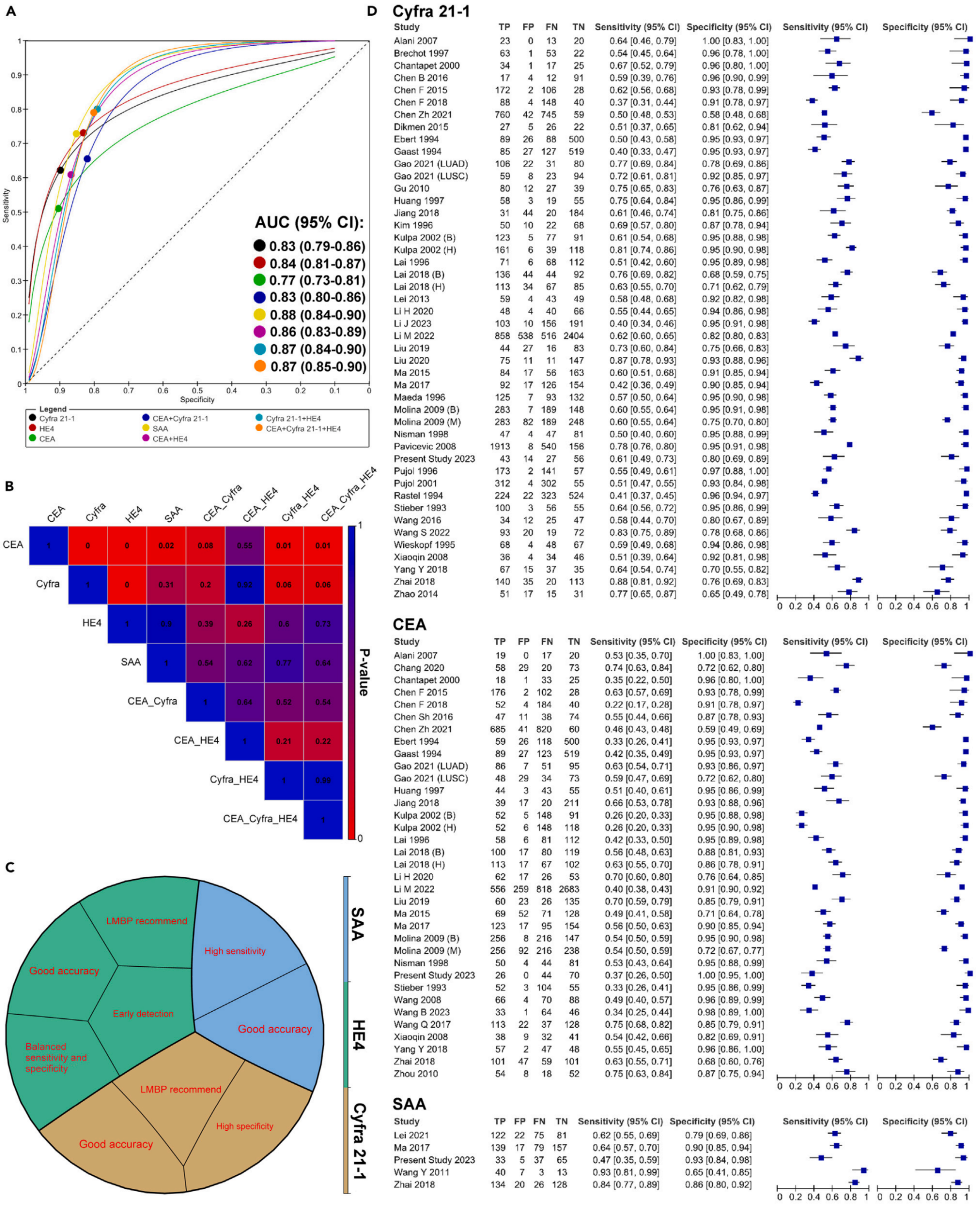
Final meta-analysis results and biomarker evaluation (A) ROC curves illustrating the diagnostic accuracy of individual biomarkers and their combinations in the final step of the meta-analysis. (B) Heatmap displaying the global test comparison *p*-value of biomarkers, highlighting the statistical significance of differences between individual biomarkers and their combinations in the final meta-analysis. (C) Overview of the key advantages of single biomarkers with good accuracy classification, as determined in the final step of the study. (D) Forest plot of Cyfra 21-1, CEA, and SAA biomarkers, summarizing sensitivity, specificity, confidence intervals, and the number of true positives (TP), false positives (FP), false negatives (FN), and true negatives (TN) for each included study. Data in forest plots are presented with 95% confidence intervals. **Abbreviations**: ROC, Receiver Operating Characteristic; CEA, Carcinoembryonic Antigen; Cyfra 21-1, Cytokeratin Fragment 21-1; HE4, Human Epididymis Protein 4; SAA, Serum Amyloid A; TP, True Positives; FP, False Positives; FN, False Negatives; TN, True Negatives.

In this step, we integrated the results of our experimental validation study with previously modified data that excluded outliers obtained from step 1 and then re-implemented the meta-analysis. The results revealed that SAA, HE4, and Cyfra 21-1 as single biomarkers have AUC >0.80, which is categorized as “good” accuracy (Figure 4A; Table 2). SAA did not demonstrate significant superiority over Cyfra 21-1 and HE4, while HE4 exhibited significantly better overall accuracy compared to Cyfra 21-1 (*p* < 0.01). Moreover, combining biomarkers did not improve the accuracy of NSCLC detection compared to those single biomarkers which had “good” accuracies (Figures 4B; Tables 2 and S7). Figure 4C summarizes the most significant advantage of single biomarkers classified as "good" based on their AUC, which is greater than 0.80, in the final step of this study.

**Table 2:** Diagnostic accuracy of biomarkers in the final step meta-analysis

	P-Se (95% CI)	P-Sp (95% CI)	PLR+ (95% CI)	PLR-(95% CI)	DOR (95% CI)	AUC (95% CI)	Bivariate I2%	TE %	PB
CEA	0.51 (0.46–0.56)	0.90 (0.87–0.93)	5.3 (4.1–6.9)	0.54 (0.49–0.60)	10 (7–13)	0.77 (0.73–0.81)	0.82	30	0.06
Cyfra 21-1	0.62 (0.58–0.66)	0.90 (0.87–0.92)	6.1 (4.9–7.7)	0.42 (0.38–0.47)	15 (11–19)	0.83 (0.79–0.86)	0.82	13	0.06
HE4	0.73 (0.68–0.77)	0.83 (0.78–0.87)	4.3 (3.4–5.5)	0.32 (0.28–0.38)	13 (10–18)	0.84 (0.81–0.87)	0.763	22	0.01
SAA	0.73 (0.54–0.86)	0.85 (0.76–0.91)	4.9 (3.3–7.2)	0.32 (0.19–0.55)	15 (8–29)	0.88 (0.84–0.90)	0.75	59	0.99
CEA+Cyfra 21	0.66 (0.51–0.78)	0.82 (0.75–0.87)	3.64 (2.43–5.44)	0.42 (0.28–0.64)	8.65 (4.10–18.22)	0.83 (0.80–0.86)	0.69	0	0.75
CEA+HE4	0.61 (0.39–0.79)	0.87 (0.76–0.93)	4.59 (2.28–9.24)	0.45 (0.26–0.78)	10.18 (3.29–31.51)	0.86 (0.83–0.89)	0.99	100	0.24
Cyfra 21-1+HE4	0.80 (0.59–0.92)	0.79 (0.67–0.88)	3.83 (2.30–6.40)	0.25 (0.11–0.58)	15.18 (4.91–46.91)	0.87(0.84–0.90)	0.62	14	0.13
CEA+Cyfra 21-1+HE4	0.79 (0.61–0.90)	0.79 (0.66–0.88)	3.81 (2.42–6.00)	0.27 (0.15–0.49)	14.16 (6.81–29.44)	0.87 (0.85–0.90)	0.71	12	0.14

CI: Confidence interval; P-Se: Pooled sensitivity; P-Sp: Pooled specificity; PLR: Pooled likelihood ratio; DOR: Diagnostic odds ratio; AUC: Area under curve; TE: Threshold effect; PB: Publication bias.

**Figure 5:**
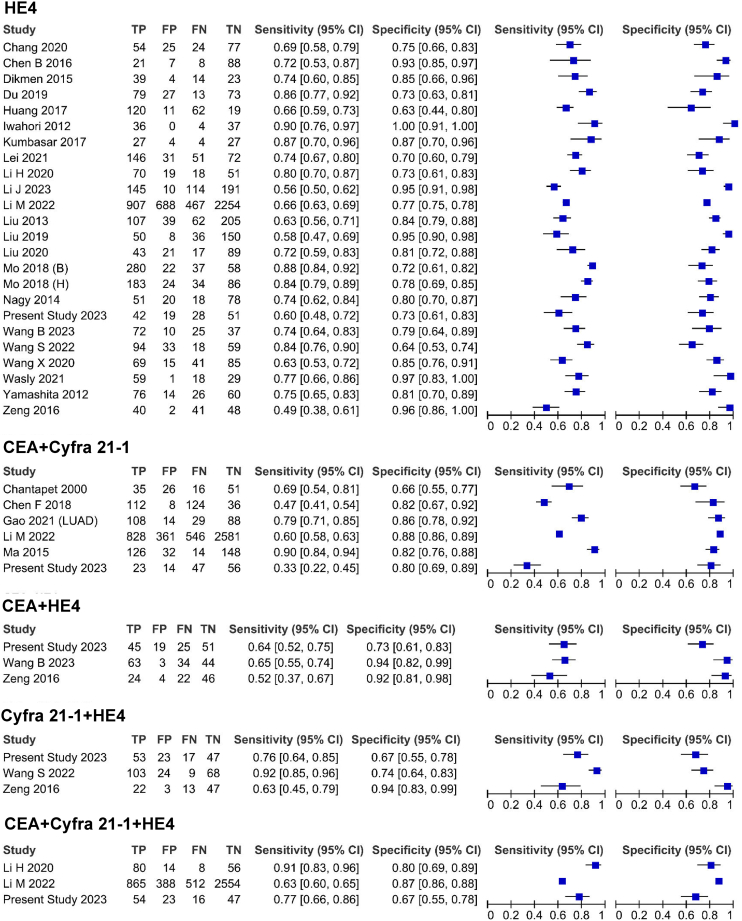
Forest plot of HE4 and combination biomarkers The forest plot shows the summary of sensitivity and specificity, along with their confidence intervals, for HE4 and biomarker combinations. The plot also details the True Positives, False Positives, False Negatives, and True Negatives reported in each included study Sensitivity and specificity are presented with 95% confidence intervals. **Abbreviations:** HE4, Human Epididymis Protein 4; CEA, Carcinoembryonic Antigen; Cyfra 21-1, Cytokeratin Fragment 21-1; TP, True Positives; FP, False Positives; FN, False Negatives; TN, True Negatives.

Bivariate I2 analysis revealed considerable heterogeneity in pooled sensitivity and specificity between studies across various groups (Figures 4D and 5; Table 2). One of the most important aims of this step was to consider a wide range of covariates as sources of heterogeneity to identify their potential confounding effects on the diagnostic accuracy of biomarkers. Subgroup analysis and assessment of threshold effects were performed to examine the possible sources of this heterogeneity. For subgroup analysis, various covariates were considered, including stage, methods of measurement, and the composition of the control group. However, the available data were insufficient to analyze age and histologic subtypes for single biomarkers, as well as all aforementioned covariates for combined biomarkers.

Regarding the subgroup analysis based on stage, the percentage of tumor stage was divided into two subgroups: studies with less than 50% in late stages compared to studies with more than 50% in late stages. Our findings indicated that higher stages significantly affected the specificity of CEA, Cyfra 21-1, and SAA (*p*-values = 0.04, 0.01, and 0.03, respectively), as well as their overall accuracy (*p*-values = 0.04, 0.04, and 0.02, respectively), but not their sensitivities. However, tumor stage had no effect on the sensitivity, specificity, or overall accuracy in the HE4 group, indicating that HE4 is a suitable biomarker for the early diagnosis of NSCLC (Table 3). Therefore, tumor stage can be considered a source of heterogeneity for CEA, Cyfra 21-1, and SAA, but not for HE4. Subgroup analysis based on methods of measurement and the composition of the control group did not show any significant impact on the performance of the evaluated biomarkers in this study. Thus, these factors cannot be deemed sources of heterogeneity (Table 3).

**Table 3:** the results of subgroup analysis in the final step meta-analysis

Analysis type	Relative Sensitivity (95% CI)	Sensitivity *p*-value	Relative Specificity (95% CI)	Specificity *p*-value	Global test comparison *p*-value
**non-CLIA vs. CLIA method**					

**CEA**	0.97 (0.78–1.21)	0.81	1.05 (0.99–1.12)	0.10	0.24
**Cyfra 21-1**	1.08 (0.92–1.27)	0.30	1.03 (0.98–1.10)	0.18	0.15
**HE4**	1.05 (0.93–1.19)	0.39	1.04 (0.93–1.15)	0.48	0.27

**Late Stages vs. Early stages**					

**CEA**	0.87 (0.66–1.15)	0.34	1.10 (1.00–1.21)	0.041	0.049
**Cyfra 21-1**	1.00 (0.86–1.16)	0.99	1.11 (1.01–1.22)	0.018	0.048
**HE4**	1.09 (0.93–1.28)	0.30	0.99 (0.87–1.12)	0.86	0.41
**SAA**	1.18 (0.72–1.93)	0.51	0.81 (0.66–0.98)	0.03	0.022

**Healthy vs. Benign controls**					

**CEA**	1.16 (0.92–1.48)	0.21	0.95 (0.89–1.02)	0.18	0.36
**Cyfra 21-1**	0.99 (0.81–1.22)	0.98	0.98 (0.91–1.06)	0.68	0.89
**HE4**	0.88 (0.76–1.00)	0.12	1.01 (0.84–1.21)	0.87	0.26

CLIA: chemiluminescence immunoassay; CI: Confidence interval.

In diagnostic accuracy studies, the threshold effect is considered one of the most crucial factors contributing to heterogeneity. Our analysis revealed that the highest proportion of heterogeneity for single biomarkers, likely due to the threshold effect, belonged to SAA, with 59% of the heterogeneity attributed to this effect. Other biomarkers exhibited varying proportions of heterogeneity likely due to the threshold effect, ranging from 13% to 30% (Table 2). For combined biomarkers, CEA+HE4 showed that 100% of its heterogeneity was likely due to the threshold effect, while other combinations, such as Cyfra 21-1+HE4 and CEA+ Cyfra 21-1+HE4 combinations, had low levels of heterogeneity likely due to the threshold effect (Table 2). Further, publication bias analysis showed that the integration of our experimental results led to the generation of publication bias compared to the initial meta-analysis in CEA. Cyfra 21-1 and HE4 still exhibited significant publication bias (Table 2).

## Discussion

This study provides a comprehensive summary and comparison of the diagnostic performance of immunoassay-based serum biomarkers for diagnosing NSCLC, employing the CDC-recommended LMBP method. This approach represents a significant advancement over previous research, which has typically focused on individual biomarkers without applying a structured, evidence-based framework such as LMBP. The LMBP method allowed us to rigorously evaluate the quality and effect size of each study, leading to more reliable and clinically relevant conclusions. To achieve this goal, we designed a three-step study: First, we conducted a systematic review and meta-analysis, implementing the CDC-recommended LMBP method, to identify clinically applicable biomarkers. Second, we validated the initial findings through direct comparisons in a case-control experimental design, addressing the limitations of indirect comparisons in the first step. Finally, we integrated these experimental results with the initial meta-analysis, which included an assessment of covariates and identification of potential confounders, to provide a more nuanced understanding of the diagnostic accuracy of the biomarkers.

The primary objective of our study was to identify the most accurate immunoassay-based serum biomarker for diagnosing NSCLC among the available options. Our results show that SAA, Cyfra 21-1, and HE4 had AUCs greater than 0.80, which were classified as “good” accuracy, all outperforming CEA. Specifically, SAA had the highest pooled sensitivity (0.71; 95% CI: 0.54–0.86), making it particularly effective at identifying true positives. Cyfra 21-1 demonstrated the highest pooled specificity (0.90; 95% CI: 0.87–0.92), indicating its strength in correctly identifying true negatives. HE4 displayed a balanced diagnostic profile with a sensitivity of 0.73 (95% CI: 0.68–0.77) and a specificity of 0.83 (95% CI: 0.78–0.87), making it a strong overall candidate for NSCLC diagnosis. While SAA did not show significant superiority over Cyfra 21-1 and HE4, HE4 demonstrated significantly better accuracy than Cyfra 21-1 (*p* < 0.01) (Figure 4B). The pattern observed in the final indirect meta-analysis closely matched the results of the direct comparisons in the second step, suggesting that the outcomes of indirect comparisons can be replicated through direct analysis. Notably, before our study, no meta-analyses had compared the accuracy of different immunoassay-based serum biomarkers for diagnosing NSCLC. However, our findings align with individual experimental studies that previously reported SAA as more accurate in detecting NSCLC than both CEA and Cyfra 21-1, regardless of histologic type.^32^^,^^43^ Similarly, our results are consistent with previous research showing that HE4 had a higher detection priority for NSCLC compared to CEA and Cyfra 21-1 across different NSCLC subtypes. Nevertheless, the significance of these differences remains undetermined.^42^^,^^83^ Another aspect of our analysis involved comparing the accuracy of combining biomarkers. Our results indicate that combining biomarkers did not enhance diagnostic performance compared to using SAA, Cyfra 21-1, or HE4 alone, both in our experimental study and final meta-analysis. However, the limited number of studies on biomarker combinations restricts the reliability of conclusions regarding their efficacy. Therefore, future studies are strongly recommended to conduct comprehensive evaluations. Importantly, our findings demonstrate that immunoassay-based biomarkers such as SAA, Cyfra 21-1, and HE4 exhibit diagnostic accuracy comparable to that of newly proposed serum biomarkers, which often require more complex methodologies and higher costs. These serum markers, including liquid exosomes,^84^ microRNAs,^85^^,^^86^ matrix-assisted laser desorption/ionization-time of flight mass spectrometry,^87^ and long non-coding RNAs,^88^ generally show “good” accuracy with AUCs ranging between 0.80 and 0.89 for NSCLC. Therefore, immunoassay-based biomarkers offer a similarly effective yet potentially more accessible and cost-efficient alternative for NSCLC diagnosis.

Cyfra21-1 was first reported in 1992 and has since been extensively studied as a biomarker for diagnosing lung cancer.^35^ Some studies have shown Cyfra 21-1 to be a promising biomarker for lung cancer, while other research papers have indicated its low diagnostic sensitivity for the disease. Our final meta-analysis showed a “good” overall accuracy with an AUC of 0.83 (95% CI: 0.79–0.86). The likelihood ratio (LR) analysis revealed that Cyfra 21-1 has a “moderate” potential for indicating the presence of the disease (Pooled-Positive-LR (PPLR) = 6.1 (95% CI: 4.9–7.7)), but provides “small” evidence for ruling out its existence (Pooled-Negative-LR (PNLR) = 0.42 (95% CI: 0.38–0.47)) (Table 2). Two previous meta-analyses assessed the diagnostic accuracy of Cyfra 21-1^4^^,^^89^ using a univariate random-effects model, which is not recommended for DTA studies.^9^^,^^90^^,^^91^^,^^92^^,^^93^ Although our study’s results are compatible with these previously published meta-analyses, it indicated the diagnostic accuracy of Cyfra 21-1 using the standard bivariate model,^9^^,^^92^^,^^93^ with a large number of included studies. Additionally, another strength of our study was assessing the different covariates on the diagnostic performance of Cyfra 21-1. Despite our experimental study results, which indicated that late stage could not affect the diagnostic accuracy of Cyfra 21-1, the final meta-analysis results showed that Cyfra 21-1 is significantly affected by tumor stage (Table 3). This discrepancy may be due to the limited number of late-stage samples compared to early-stage samples in our experimental study, which likely influenced the results. This serves as a good example of why individual studies are not suitable for clinical decision-making and why clinical decisions should be based on evidence-based meta-analyses that include a large number of samples. In accordance with our final meta-analysis findings, previous individual reports have shown a positive correlation between serum levels of Cyfra 21-1 and tumor stage.^58^^,^^59^^,^^62^^,^^94^ Furthermore, our experimental study assessed additional covariates, such as age and histologic subtypes of NSCLC, that were not amenable to subgroup analysis due to data constraints. Our findings clearly demonstrated a positive correlation between older age and the accuracy of Cyfra21-1 in detecting NSCLC (Figure 3G). Additionally, we observed a lower accuracy of Cyfra21-1 in LUAD subtype compared to LUSC and LLCC subtypes (Figures 3F and 3G), which aligns with the results of previous studies.^23^^,^^59^ Furthermore, we found no evidence of any effect of other covariates on the diagnostic performance of Cyfra 21-1.

HE4, a promising biomarker, has been widely utilized in the diagnosis of various malignant tumors, particularly ovarian cancer. Additionally, accumulating evidence suggests the potential of HE4 in diagnosing lung cancer. The final meta-analysis demonstrated “good” overall accuracy with an AUC of 0.84 (95%CI: 0.81–0.87). Moreover, our LR analysis indicated that HE4 provides “small” evidence to both confirm and rule out the disease (PPLR = 4.30 (95% CI: 3.4–5.5) and PNLR = 0.32 (95% CI: 0.28–0.38)). While three meta-analyses have been published on the diagnostic accuracy of HE4 in lung cancer,^5^^,^^6^^,^^95^ only one of them reports the accuracy of HE4 specifically in patients with NSCLC , with a subgroup analysis including only 7 papers.^5^ Despite the limited number of studies included, our results are consistent with their findings. Their study also identified the influence of laboratory method on the diagnostic performance of HE4, but due to the analysis being conducted on all patients with lung cancer, it is not directly comparable to our study. Furthermore, there is a lack of previous pooled data regarding the impact of different covariates on HE4 diagnostic performance. Our experimental and final meta-analysis results demonstrate similar diagnostic performance of HE4 in both early and late stages of NSCLC. These results suggest that HE4 has “good” accuracy for the early diagnosis of NSCLC, which is supported by several studies.^14^^,^^28^^,^^53^ Given its high diagnostic accuracy and consistent performance across different stages of NSCLC, HE4 has significant potential for integration into clinical practice. Utilizing HE4 in diagnostic protocols could enhance the early detection of NSCLC, leading to more timely interventions and improved patient outcomes. Reliability of HE4, irrespective of tumor stage, supports its use in routine screenings and diagnostic assessments. Therefore, HE4 should be considered for inclusion in clinical guidelines, alongside other established markers such as Cyfra 21-1 and CEA. However, in contrast to our experimental and meta-analysis results, some individual studies have reported a positive correlation between serum levels of HE4 and advanced tumor stages.^28^^,^^29^^,^^46^^,^^67^ These discrepancies highlight the complex nature of biomarker performance across different stages and patient populations. These findings emphasize the importance of making clinical decisions based on comprehensive meta-analyses rather than individual experimental studies. Our experimental study evaluated additional covariates, such as age and histologic subtypes of NSCLC, which were not included in the subgroup analysis due to limited data availability. Our experiments revealed a correlation between higher age and increased diagnostic accuracy for NSCLC. Moreover, we found that LUSC and LLCC had higher accuracy in detecting NSCLC compared to LUAD (Figures 3F and 3G). The studies conducted by Li et al.^28^ and Nagy et al.^71^ showed similar findings in this regard. However, Zeng et al.^42^ did not find any significant differences in the accuracy of HE4 for detecting LUAD or LUSC.

SAA, a type of cytokine-induced acute inflammatory response protein, has been implicated in cancer development.^96^ Our meta-analysis results revealed that SAA is the most accurate biomarker for detecting NSCLC, with an overall AUC of 0.88 (95%CI: 0.84–0.90), indicating a "good" overall accuracy. Additionally, our LR analysis demonstrated that SAA provides "small" evidence both for confirming and ruling out the presence of the disease (PPLR = 4.9 (95% CI: 3.3–7.9) and PNLR = 0.32 (95% CI: 0.19–0.55)). Similar to Cyfra 21-1, our experimental analysis showed that tumor stage does not affect the diagnostic accuracy of SAA for NSCLC. However, subgroup analysis from the final meta-analysis indicated that higher tumor stages positively affect the diagnostic accuracy of SAA, which aligns with findings from individual studies.^68^ The possible reason of discrepancy between our experimental step and final meta-analysis results in regard of the effect of tumor stage is limited number of late-stage samples. The discrepancy between our experimental results and the final meta-analysis regarding the effect of tumor stage is likely due to the limited number of late-stage samples in the experimental step. This discrepancy underscores the importance of basing clinical decisions on evidence-based meta-analyses, which consider larger and more diverse sample sets, rather than relying solely on individual studies. Despite the "good" accuracy of SAA, it is crucial to consider that SAA is an acute-phase protein that increases in various inflammatory conditions and is not highly specific to malignancy. This lack of specificity may lead to an increased rate of false positives, particularly when used in non-malignant patients with underlying inflammatory conditions. Moreover, our experimental study assessed additional covariates, including age and histologic subtypes of NSCLC, which were not included in the subgroup analysis due to limited data availability. In this context, older age was correlated with higher diagnostic accuracy for NSCLC. Additionally, SAA demonstrated higher accuracy in detecting LUSC and LLCC compared to LUAD (Figures 3F and 3G).

In conclusion, this study, utilizing a comprehensive evidence-based approach through the LMBP framework, determines that HE4 is the most reliable serum biomarker for the early detection of NSCLC and the leading candidate for routine clinical use. This conclusion is supported by the finding that, while Cyfra 21-1 and SAA also demonstrated “good” accuracy, HE4 consistently outperformed them, particularly in early-stage detection, showing significant superiority over Cyfra 21-1 and offering a more balanced sensitivity and specificity. The findings of this study suggest that integrating HE4 into current NSCLC diagnostic protocols could enhance early detection, reduce the need for invasive procedures, and potentially lower healthcare costs. As such, we recommend that HE4 be considered for inclusion in clinical guidelines for NSCLC diagnosis, alongside other established biomarkers such as Cyfra 21-1 and CEA.

### Limitations of the study

Despite strengths of the present study, there are limitations that should be considered when interpreting our findings. First, most of the included studies followed a case-control design, which introduces potential bias in patient selection. To address this limitation, we constructed multiple contingency tables, where possible, comparing specific characteristics not only with healthy controls but also with other patients lacking those specific characteristics. Additionally, we included both healthy individuals and patients with benign lung diseases as control groups in our experimental validation to minimize selection bias. Secondly, in the final meta-analysis we encountered difficulties in calculating the impact of certain covariates for some biomarkers due to the unavailability of sufficient raw data. Specifically, many of the included studies did not provide enough detailed data to construct the 2x2 contingency tables required for bivariate meta-regression analysis. Consequently, we were unable to fully assess how these covariates influence biomarker performance in the meta-analysis. To mitigate this limitation, we evaluated these covariates in our experimental study. However, findings based on this individual experimental data should be interpreted with caution until confirmed by future meta-analyses. Thirdly, the limited number of studies that included biomarker combinations restricted our ability to draw definitive conclusions on the diagnostic performance of these combinations. Finally, we identified the presence of publication bias concerning CEA, Cyfra 21-1, and HE4, indicating a potential overestimation of the diagnostic performance of these biomarkers, as studies with higher DOR results were more likely to be published.

## Resource availability

### Lead contact

Further information and requests for resources and reagents should be directed to and will be fulfilled by the lead contact, Jun Zhang (jameszhang2000@zju.edu.cn).

### Materials availability

This study did not generate new unique reagents.

### Data and code availability

• All data reported in this paper will be shared by the lead contact upon request.
• This study did not report original code.
• Any additional information required to reanalyze the data reported in this paper is available from the lead contact upon request.

## Acknowledgments

This work was supported by grants from the 10.13039/501100001809National Natural Science Foundation of China (82172362 and 81972012), 10.13039/100022963Key Research and Development Program of Zhejiang Province (2022C03037), 10.13039/501100004731Natural Science Foundation of Zhejiang Province (LY24H200001 and LY22H200002), Health Bureau Foundation of Zhejiang Province (2024KY1104) and Key Laboratory of Precision Medicine in Diagnosis and Monitoring Research of Zhejiang Province (2022E10018).

## Author contributions

A.N.K., M.E.Z. and J.Z. conceived and designed the project. A.N.K and M.E.Z reviewed literatures and extracted the data. A.N.K. and M.E.Z. performed statistical analysis and wrote the article. X.W, J.L, J.H, Y.W, Y.M, Y.G, performed experimental step, J.Z. reviewed the article and provided suggestions for further development. All performances were conducted under supervision of J.Z.

## Declaration of interests

The authors have no conflict of interest to disclose.

## STAR★Methods

### Key resources table

**Table:** 

REAGENT or RESOURCE	SOURCE	IDENTIFIER
**Biological samples**		

serum samples	This paper	N/A

**Critical commercial assays**		

CEA chemiluminescence assay kit	Abbott ARCHITECT	7K68-27
Cyfra 21-1 electrochemiluminescence immunoassay kit	Cobas 8000 e801	11820966122
HE4 electrochemiluminescence immunoassay kit	Roche Elecsys	05950929190
SAA assay kit	Zhejiang Zhuoyun Biotechnology	20181026

**Deposited data**		

MEDLINE/PubMed	United States National Library of Medicine	pubmed.ncbi.nlm.nih.gov
Web of Science	Clarivate	https://clarivate.com/products/scientific-and-academic-research/research-discovery-and-workflow-solutions/webofscience-platform/
Scopus	Elsevier	www.scopus.com
China National Knowledge Infrastructure (CNKI)	Tongfang Knowledge Network Technology Co., Ltd.	https://cnki.net/
Scientific Information Database (SID)	Academic Center for Education, Culture and Research	https://sid.ir/journal/en

**Software and algorithms**		

SPSS 21	IBM	N/A
STATA 14	Stata Corporation	https://www.stata.com/order/new/edu/profplus/student-pricing/
GraphPad Prism	GraphPad	https://www.graphpad.com/
Meta-DiSc 2	Plana et al.^97^	https://ciberisciii.shinyapps.io/MetaDiSc2/
RevMan 5.4	Cochrane	https://training.cochrane.org/online-learning/core-software/revman
SRplot online tool	Tang et al.^98^	https://www.bioinformatics.com.cn/srplot
MedCalc	MedCalc Software	https://www.medcalc.org/calc/comparison_of_independentROCtest.php (Version 23.0.2; accessed September 24, 2024)
R language	The Comprehensive R Archive Network	https://cran.r-project.org/
EndNote V9	Clarivate	endnote.com

### Experimental model and study participant details

#### Participants

We employed a census-based sampling approach to select 70 patients with non-small cell lung cancer (NSCLC) and 70 control individuals. The control group comprised 35 healthy volunteers and 35 individuals with non-malignant lung disorders (NMLD), including chronic obstructive pulmonary disease and infectious lung diseases, who were referred to Sir Run Run Shaw Hospital at Zhejiang University, Hangzhou, China. Participants were matched on a one-to-one basis by sex and age (within a ±2-year range) to minimize potential confounding variables and ensure comparability between groups. Recruitment took place between November 2023 and April 2024. All NSCLC and NMLD patients were untreated, with diagnosis and staging confirmed through standard pathological and radiological procedures.

#### Ethical statement

The study was conducted in accordance with the Declaration of Helsinki and received ethical approval from the Sir Run Run Shaw Hospital ethics committee (Permit number: 20230422). Informed consent was obtained from all participants before sample collection.

### Method details

This research was conducted in three systematic steps, each designed to progressively refine the identification of the most accurate immunoassay-based serum biomarkers for the diagnosis of NSCLC.

#### Step 1: Systematic review and meta-analysis

##### Search strategy

Following Preferred Reporting Items for Systematic reviews and Meta-Analyses (PRISMA) guidelines,^99^ a comprehensive search was conducted across databases including Web of Science, Scopus, MEDLINE/PubMed, Chinese National Knowledge Infrastructure (CNKI) database for Chinese full-text articles and the Scientific Information Database (SID) for Persian full-text articles up to July 20, 2023, without language restrictions. In MEDLINE/PubMed, controlled MeSH terms such as (“Carcinoma Non-Small-Cell Lung”) AND (“Diagnosis” OR “Immunologic Tests”) were used. In the other databases, we utilized free-text terms such as ("Lung Cancer" OR "Non-Small Cell Lung Cancer" OR "Lung Malignancy") AND ("Serum biomarker" OR "Laboratory Test" OR "Diagnostic biomarker"). To ensure a comprehensive search, we also reviewed the references of selected papers, examined related systematic and narrative reviews, and explored similar papers suggested by PubMed and Google Scholar. Duplicate records were removed using EndNote software (Version X9, Thomson Reuters).

##### Study selection and data extraction

Two independent reviewers (M.E.Z and A.N.K) screened the titles and abstracts of all retrieved records to determine their eligibility for inclusion. Due to the large number of biomarkers and studies, the screening process was divided into two stages. The inclusion criteria were: (1) patients with NSCLC for whom a serum immunoassay-based biomarker was used for diagnosis; (2) NSCLC confirmed by pathology reference standards; (3) sufficient diagnostic data to construct a 2 × 2 contingency table; and (4) at least four studies available for each serum biomarker. The exclusion criteria included: (1) duplicated studies, review articles, editorials, case reports, and clinical guidelines; (2) inadequate data reporting to construct the 2 × 2 contingency table; (3) NSCLC not verified by the specified reference standards; and (4) biomarkers used solely for the diagnosis of relapse or treatment response.

Following the first screening step, biomarkers without clinical application were excluded. Clinical application was defined as having more than 50% of studies related to a specific biomarker reporting an AUC >0.70 and/or an LR+ > 2 and an LR- < 0.5.

Data were extracted using a customized data extraction form, which included details such as the first author’s name, publication year, country, study design, total sample size, and diagnostic outcomes (true positives, false positives, true negatives, and false negatives). For case-control studies, 2 × 2 contingency tables were constructed by comparing characteristics not only with healthy controls but also with other patients who did not have the specific characteristics, to ensure more reliable results.

The late-stage tumors were defined as NSCLC stages III and IV, while the early-stage tumors were categorized as stage 0, I, and II. Additionally, the histologic type of NSCLC was categorized into LUAD, LCCL, and LUSC.

##### Quality assessment and publication bias

The methodological quality of each study included in the analysis was evaluated using the QUADAS-2 tool. QUADAS-2 examines four key domains: "patient selection," "index test," "reference standard," and "flow and timing." These domains are assessed across two categories: "risk of bias" for all four domains and "applicability" for the first three domains. Based on predefined assessment criteria, each category was rated as low, high, or unclear risk.^100^ Any disagreements during the assessment were resolved through consensus after discussion. Outlier studies were identified using a standardized predicted random effects scatterplot (standardized level-2 residuals). To assess potential publication bias, Deeks' funnel plot asymmetry was evaluated using a linear regression method. A *p*-value <0.1 for the slope coefficient was considered indicative of publication bias.

Implementation of Laboratory Medicine Best Practices**:** To conduct a systematic review and develop evidence-based recommendations, we employed the "Apprizing" and "Analyzing" steps of the LMBP method. The Apprizing step involves evaluating and categorizing individual studies based on their "quality" and "effect size", as previously described.^11^^,^^12^^,^^101^^,^^102^^,^^103^ Briefly, the quality of each study was assessed using a rating scale: a score of 1 for low risk of bias, 0.5 for unclear risk, and 0 for high risk of bias, assigned to each QUADAS-2 domain. Quality ratings were classified as good if the score was greater than 5, fair if the score was between 3.5 and 5, and poor if the score was less than or equal to 3.

The effect size of each study was determined using LR+ and LR-cutoffs. A substantial effect size was assigned if LR+ > 10 and LR- < 0.1. A moderate effect size was assigned if LR+ > 10 and LR- > 0.1, or if LR+ < 10 and LR- < 0.1. A minimal effect size was assigned if LR+ < 10 and LR- > 0.1.

The “Analyzing” step involves summarizing the results obtained from the “Apprizing” step within each test group to determine the overall strength of the body of evidence. The overall strength of the evidence was classified as “High” if there were 3 or more studies with a substantial effect size and good quality; “Moderate” if there were 2 studies with a substantial effect size and good quality or 3 or more studies with a moderate effect size and good quality; “Suggestive” if there was 1 study with a substantial effect size and good quality, 2 studies with a moderate effect size and good quality, or 3 or more studies with a moderate effect size and fair quality; and insufficient for any other situation.

Finally, the overall strength of the evidence was used to inform one of three evidence-based recommendations: "recommend", "no recommendation for or against due to insufficient evidence", or "recommend against". If the overall strength of the evidence was either high or moderate, the practice was rated as "recommend". When the overall strength of the evidence was suggestive or insufficient, the practice was rated as "no recommendation for or against due to insufficient evidence". The categorization of "recommend against" is reserved for situations where there is evidence of adverse effects, which was not applicable to our study.

##### Interpretation of diagnostic accuracy

The overall diagnostic accuracy of each biomarker was assessed based on the AUC, PPLR, and PNLR. Specifically, the AUC value was interpreted as follows: 0.5–0.70 (not acceptable), 0.71–0.79 (acceptable), 0.80–0.89 (good), and 0.90–1 (excellent). In addition to the AUC, the diagnostic accuracy of each biomarker was categorized using the PPLR and PNLR. PNLR values <0.1, 0.1–0.2, 0.2–0.5, and >0.5 were regarded as representing substantial, moderate, small, and nonmeaningful evidence, respectively, to rule out the existence of the disease. Similarly, PPLR values >10, 5–10, 2–5, and <2 were considered to provide substantial, moderate, small, and not meaningful evidence, respectively, to suggest the presence of the disease.

#### Step 2: Case-control validation

Based on the results from the first step, biomarkers classified as “recommend” by the LMBP method, along with the most accurate ones where relevant, were subjected to experimental evaluation through a case-control study to validate their diagnostic accuracy in a direct comparison setup. Peripheral blood samples were collected from each participant in the morning after overnight fasting. The samples were centrifuged at 3000 rpm for 10 min, and the serum was collected and immediately stored at −80°C until further analysis.

Selected biomarkers were measured using validated chemiluminescence immunoassay (CLIA) method. Pre-specified thresholds from the kit manuals were applied to all biomarkers, with independent and blinded evaluations of the reference standards and index tests. Specifically, CEA was analyzed on the Abbott ARCHITECT i4000SR analyzer with a predefined cut-off of <5.00 ng/mL; Cyfra 21-1 and HE4 were analyzed on the Cobas 8000 e801 analyzer with cut-offs of <3.3 μg/L and <70 pmol/L, respectively; and SAA levels were measured using the Beckman Coulter AU5800 clinical chemistry analyzer, with levels below 10.0 mg/L considered negative. All analyses were performed according to the manufacturers' instructions, with rigorous quality control measures in place.

#### Step 3: Meta-analysis incorporating experimental data

Experimental results from Step 2 were integrated into the meta-analysis from Step 1 to refine and reassess the diagnostic accuracy of the biomarkers. This step involved evaluating the effect of covariates as potential sources of heterogeneity on the diagnostic accuracy, using subgroup analysis and/or meta-regression. Specifically, we aimed to identify confounders that could affect the efficacy of diagnostic accuracy.

### Quantification and statistical analysis

In the first step, in order to construct a 2 × 2 contingency table, true positives, false positives, true negatives and false negatives were extracted for each included study. The summary points, which included pooled sensitivity, pooled specificity, PPLR, PNLR, and pooled diagnostic odds ratio (PDOR), were calculated using a standard bivariate method. Furthermore, a hierarchical model was employed to plot summary receiver operating characteristic (HSROC) curves, enabling the determination of the AUC as an overall measure of test performance. In this step, we employed standard bivariate meta-regression to compare the diagnostic accuracy of different biomarkers across studies, assessing relative sensitivity, relative specificity, their *p*-values, and the global test comparison *p*-value as the overall result. Calculations were conducted and summarized for reporting utilizing 95% confidence interval. Statistical significance was defined when *p* < 0.05 (except for investigating publication bias which was *p* < 0.1). Statistical analyses of this step were performed using the "midas" commands in Stata software (Stata Corporation, College Station, TX, USA, version 14.0) and Meta-DiSc-2.^97^ Furthermore, RevMan 5.4 were employed to generate comparative HSROC plots.

In the second step, to compare the expression levels of biomarkers between the control group (*n* = 70) and NSCLC patients (*n* = 70), we utilized a paired t-test (or, when applicable, its non-parametric equivalent, the Wilcoxon test) and visualized the results using a connection line plot to show each NSCLC patient and its matched control sample. To determine the diagnostic accuracy of the combination biomarkers, a logistic regression model was employed. To compare the diagnostic accuracy of single and combined biomarkers, we utilized AUC comparison, which was reported by Hanley & McNeil^104^^,^^105^ and implemented in the MedCalc online tool using the Comparison of AUC of Independent ROC Curves feature. Statistical analyses were performed using IBM SPSS (version 21), the SRplot online tool,^98^ and GraphPad Prism (version 8.5), with a significance level set at *p* < 0.05. Throughout this step, we strictly followed the Standards for Reporting of Diagnostic Accuracy (STARD) guidelines.^106^

In the third step, statistical analyses were conducted the same as the first step, while significant heterogeneity was indicated by Higgins' I^2^ > 50%, guiding further investigation into how different covariates might influence the results. Subgroup analysis and/or meta-regression for assessing the effect of covariates were performed by standard bivariate meta-regression, evaluating relative sensitivity, relative specificity, their *p*-values, and the global test comparison *p*-value as the overall result. The threshold effect, considered one of the key contributors to heterogeneity, was quantified as the percentage of total heterogeneity likely due to this effect.
