# Supplementary material for: Serum human epididymis Protein-4 outperforms conventional biomarkers in the early detection of non-small cell lung cancer
Source: iScience. 2024 Oct 19;27(11):111211. doi: 10.1016/j.isci.2024.111211 (PMC11550588; doi:10.1016/j.isci.2024.111211)
Supplement: Document S1. Tables S1–S7 [file mmc1.pdf]

iScience, Volume *27*

Supplemental information

Serum human epididymis Protein-4 outperforms conventional biomarkers in the early detection of non-small cell lung cancer

Mohammad Erfan Zare, Atefeh Nasir Kansestani, Xuanlan Wu, Lin Zhou, Jie Lu, Jun Huang, Yanzhong Wang, Yilei Ma, Yuzhen Gao, and Jun Zhang

Table S1. Diagnostic accuracy of biomarkers in the first step of study.

Test	P-Se (95% CI)	P-Sp (95% CI)	PLR+ (95% CI)	PLR- (95% CI)	DOR (95% CI)	AUC (95% CI)	Bivariate I2 %	TE %	PB
**Individual biomarker accuracy**									
**Initial biomarker accuracy**									
**CEA**	0.50 (0.45-0.55)	0.90 (0.86-0.92)	4.84 (3.68-6.37)	0.56 (0.50-0.61)	8.71 (6.36-11.91)	0.76 (0.72-0.80)	85.6	21%	0.15
**Cyfra 21-1**	0.63 (0.58-0.68)	0.90 (0.87-0.92)	6.13 (4.80-7.82)	0.41 (0.36-0.47)	14.94 (10.96-20.36)	0.85 (0.82-0.88)	86.9	7%	0.06
**HE4**	0.75 (0.70-0.79)	0.83 (0.78-0.87)	4.33 (3.42-5.50)	0.31 (0.26-0.36)	14.14 (10.6-18.88)	0.85 (0.82-0.88)	76.6	36%	<0.0 1
**PK-M2**	0.72 (0.68-0.75)	0.89 (0.78-0.95)	6.38 (3.15-12.91)	0.32 (0.28-0.37)	19.924 (8.97-44.23)	0.73 (0.69-0.77)	63.2	100%	0.67
**SAA**	0.78 (0.60-0.89)	0.83 (0.73-0.90)	4.56 (3.01-6.91)	0.27 (0.15-0.49)	16.98 (8.08-35.69)	0.88 (0.84-0.90)	75.2	36%	0.93
**VEGF**	0.69 (0.58-0.77)	0.86 (0.78-0.92)	5.04 (2.92-8.70)	0.36 (0.26-0.51)	13.86 (6.13-31.35)	0.85 (0.81-0.88)	83.4	13%	0.76
**Biomarker accuracy after implementing laboratory medicine best practice method and conducting outlier exclusion**									
**CEA**	0.51 (0.46-0.57)	0.90 (0.87-0.92)	5.08 (3.95-6.52)	0.54 (0.49-0.59)	9.40 (7.07-12.50)	0.78 (0.74-0.81)	83.1	21%	0.15
**Cyfra 21-1**	0.62 (0.58-0.66)	0.90 (0.87-0.92)	6.23 (4.93-7.87)	0.42 (0.37-0.46)	14.85 (11.31-19.51)	0.83 (0.79-0.86)	82.2	7%	0.06
**HE4**	0.74 (0.69-0.78)	0.84 (0.79-0.87)	4.45 (3.48-5.69)	0.31 (0.27-0.37)	14.03 (10.37-18.99)	0.85 (0.81-0.88)	75.3	36%	<0.0 1
**Biomarker Combination accuracy**									
**CEA+Cyfra 21-1**	0.71 (0.57-0.81)	0.82 (0.76-0.87)	4.05 (2.83-5.79)	0.35 (0.22-0.54)	11.54 (5.70-23.33)	-	-	-	-
**CEA+HE4**	0.59 (0.35-0.79)	0.93 (0.84-0.97)	8.50 (3.32-21.77)	0.44 (0.24-0.78)	19.35 (5.14-72.83)	-	-	-	-
**Cyfra 21-1+HE4**	0.81 (0.57-0.93)	0.84 (0.70-0.93)	5.42 (2.74-10.71)	0.23 (0.8-0.55)	25.50 (7.61-85-44)	-	-	-	-
**CEA+Cyfra 21-1+HE4**	0.79 (0.55-0.92)	0.84 (0.75-0.90)	5.06 (3.22-7.95)	0.24 (0.09-0.59)	20.88 (6.77-64.38)	-	-	-	-

CEA: Carcinoma embryonic antigen; Cyfra 21-1: Cytokeratin 19-fragments; HE4: Human epididymis protein-4; SAA: Serum amyloid-A; CI: Confidence interval; P-Se: Pooled sensitivity; P-Sp: Pooled specificity; PLR: Pooled likelihood ratio; DOR: Diagnostic odds ratio; AUC: Area under curve; TE: Threshold effect; PB: Publication bias

Table S2. Comparison biomarker accuracy in the first step of study.

Test	Relative Sensitivity	95% CI	Sensitivity p-value	Relative Specificity	95% CI	Specificity p-value	Global test comparison
**Initial Comparison**							
**Cyfra 21-1 vs. CEA**	1.25	1.09-1.44	<0.001	1.001	0.95-1.04	0.96	<0.01
**HE4 vs. CEA**	1.49	1.32-1.68	<0.001	0.93	0.86-0.99	0.03	<0.001
**PK-M2 vs. CEA**	1.44	1.23-1.69	<0.001	0.988	0.90-1.08	0.78	<0.01
**SAA vs. CEA**	1.53	1.26-1.85	<0.01	0.919	0.77-1.08	0.22	<0.01
**VEGF vs. CEA**	1.37	1.14-1.64	<0.01	0.96	0.87-1.06	0.42	0.01
**HE4 vs. Cyfra 21-1**	1.19	1.07-1.32	<0.01	0.93	0.87-0.99	0.02	<0.01
**PK-M2 vs. Cyfra 21-1**	1.14	0.97-1.35	0.15	0.98	0.90-1.07	0.77	0.35
**SAA vs. Cyfra 21-1**	1.22	1.01-1.48	0.09	0.92	0.78-1.08	0.20	0.17
**VEGF vs. Cyfra 21-1**	1.09	0.90-1.32	0.39	0.96	0.87-1.06	0.41	0.55
**PK-M2 vs HE4**	0.97	0.85-1.09	0.62	1.06	0.96-1.16	0.26	0.53
**SAA vs. HE4**	1.02	0.87-1.20	0.76	0.99	0.86-1.14	0.96	0.94
**VEGF vs. HE4**	0.91	0.78-1.07	0.25	1.03	0.94-1.14	0.47	0.48
**SAA vs. PK-M2**	1.03	0.90-1.19	0.60	0.93	0.78-1.10	0.38	0.65
**VEGF vs. PK-M2**	0.94	0.82-1.09	0.44	0.97	0.86-1.10	0.70	0.72
**VEGF vs. SAA**	0.89	0.72-1.11	0.34	1.03	0.90-1.18	0.57	0.54
**Comparison after implementing laboratory medicine best practice method and conducting outlier exclusion**							
**Cyfra 21-1 vs. CEA**	1.75	1.49 -2.04	<0.001	0.67	0.60-0.76	<0.001	<0.001
**HE4 vs. CEA**	1.43	1.27-1.60	<0.001	0.93	0.87-0.99	0.02	<0.001
**HE4 vs. Cyfra 21-1**	1.18	1.08-1.29	<0.001	0.93	0.87-0.99	0.01	<0.01
**SAA vs. CEA**	1.49	1.24-1.78	<0.01	0.91	0.78-1.06	0.16	<0.01
**SAA vs. Cyfra 21-1**	1.23	1.05-1.44	0.03	0.91	0.78-1.06	0.15	0.08
**SAA vs. HE4**	1.03	0.89-1.20	0.64	0.98	0.86-1.13	0.86	0.89
**CEA+Cyfra 21-1 vs. CEA**	1.37	1.13-1.67	<0.01	0.91	0.80-1.04	0.11	0.03
**CEA+HE4 vs. CEA**	1.15	0.79-1.66	0.48	1.03	0.94-1.14	0.52	0.48
**Cyfra 21-1+HE4 vs. CEA**	1.59	1.30-1.95	<0.01	0.96	0.81-1.14	0.65	0.01
**CEA+Cyfra 21-1+HE4 vs. CEA**	1.52	1.22-1.89	0.01	0.93	0.76-1.12	0.36	0.03
**CEA+Cyfra 21-1 vs. Cyfra 21-1**	1.13	0.96-1.35	0.18	0.91	0.80-1.04	0.10	0.18
**CEA+HE4 vs. Cyfra 21-1**	0.95	0.67-1.34	0.77	1.03	0.94-1.14	0.53	0.81
**Cyfra 21-1+HE4 vs. Cyfra 21-1**	1.31	1.10-1.57	0.03	0.96	0.80-1.14	0.62	0.11
**CEA+Cyfra 21-1+HE4 vs. Cyfra 21-1**	1.25	1.03-1.53	0.07	0.93	0.76-1.12	0.36	0.20
**CEA+Cyfra 21-1 vs. HE4**	0.96	0.81-1.13	0.63	0.98	0.87-1.11	0.83	0.81
**CEA+HE4 vs. HE4**	0.80	0.59-1.10	0.11	1.11	1.01-1.22	0.11	0.17
**Cyfra 21-1+HE4 vs. HE4**	1.17	0.94-1.32	0.28	1.03	0.88-1.21	0.68	0.35
**CEA+Cyfra 21-1+HE4 vs. HE4**	1.05	0.87-1.27	0.58	1.00	0.85-1.17	0.98	0.82
**CEA+Cyfra 21-1 vs. SAA**	0.95	0.76-1.20	0.69	1.12	0.83-1.51	0.43	0.73
**CEA+HE4 vs. SAA**	1.19	0.98-1.44	0.13	0.74	0.58-0.94	0.03	0.10
**Cyfra 21-1+HE4 vs. SAA**	1.10	0.88-1.37	0.42	1.01	0.87-1.16	0.88	0.64
**CEA+Cyfra 21-1+HE4 vs. SAA**	1.00	0.77-1.30	0.97	1.00	0.90-1.11	0.96	0.99
**CEA+HE4 vs. CEA+Cyfra 21-1**	0.83	0.53-1.28	0.37	1.12	1.02-1.24	0.04	0.10
**Cyfra 21-1+HE4 vs. CEA+Cyfra 21-1**	1.14	0.84-1.55	0.42	1.02	0.88-1.20	0.72	0.57
**Cyfra 21-1+HE4 vs. CEA+HE4**	1.37	0.90-2.1	0.16	0.92	0.79-1.07	0.33	0.37
**CEA+Cyfra 21-1+HE4 vs. CEA+Cyfra 21-1**	1.11	0.81-1.52	0.52	1.02	0.91-1.13	0.72	0.70
**CEA+Cyfra 21-1+HE4 vs. CEA+HE4**	1.33	0.87-2.03	0.18	0.90	0.83-0.98	0.02	0.09
**CEA+Cyfra 21-1+HE4 vs. Cyfra 21-1+HE4**	0.96	0.66-1.39	0.84	0.98	0.83-1.16	0.85	0.86

CEA: Carcinoma embryonic antigen; Cyfra 21-1: Cytokeratin 19-fragments; HE4: Human epididymis protein-4; SAA: Serum amyloid-A; NSCLC: Non-small-cell lung cancer; LUAD: Lung adenocarcinoma; LUSC: Lung squamous cell carcinoma; LLCC: Lung large cell carcinoma; CI: Confidence interval; P-Se: Pooled sensitivity; P-Sp: Pooled specificity; PLR: Pooled likelihood ratio; DOR: Diagnostic odds ratio; AUC: Area under curve; TE: Threshold effect; PB: Publication bias.

Table S3. The comparison of accuracies after and before outlier exclusion in the first step of study.

Biomarker	Relative Sensitivity (95% CI)	Sensitivity p-value	Relative Specificity (95% CI)	Specificity p-value	Global test comparison
CEA	1.02 (0.88-1.18)	0.72	1.00 (0.96-1.05)	0.83	0.87
Cyfra 21-1	0.98 (0.89-1.09)	0.81	1.00 (0.96-1.04)	0.80	0.95
HE4	0.98 (0.90-1.07)	0.73	1.00 (0.93-1.08)	0.82	0.94

CEA: Carcinoma embryonic antigen; Cyfra 21-1: Cytokeratin 19-fragments; HE4: Human epididymis protein-4; SAA: Serum amyloid-A; CI: Confidence interval.

Table S4. Characteristic of included patients in experimental validation step of study.

		NSCLC (n=70)			Control (n= 70)	
		**LUAD**	**LLCC**	**LUSC**	**NMLD**	**Healthy controls**
**Subtype, n (%)**		34 (48.57)	3 (4.28)	33 (47.14)	35 (50)	35 (50)
**Age, mean ± SD**		59.71 ± 10.46	69.33 ± 8.26	65.33 ± 9.40	65.75 ± 9.05	59.82 ± 10.68
**Gender, n (%)**	**Male**	12 (35.3)	3 (100)	32 (97)	23	24
	**Female**	22 (64.7)	0 (0)	1 (3)	12	11
**Stage*, n (%)**	**Early Stages (n= 63)**	29 (85.29)	3 (100)	31 (93.9)	-	-
	**Late Stages (n= 7)**	5 (14.7)	0 (0)	2 (6)	-	-

NSCLC: Non-small-cell lung cancer; LUAD: Lung adenocarcinoma; LUSC: Lung squamous cell carcinoma; LLCC: Lung large cell carcinoma; NMLD: non-malignant lung disorders. * Significant difference; $P<0.05$

Table S5. Diagnostic accuracy of biomarkers in total cases and controls, and by covariates, in the experimental validation step of the study.

**Biomarker**		**Sensitivity (95% CI)**	**Specificity (95% CI)**	**AUC**
CEA	All controls vs. all NSCLC	37.14 (25.89-49.52)	100 (94.87-100)	0.72
	Benign controls vs. all NSCLC	37.14 (25.89-49.52)	100 (87.66-100.00)	0.69
	Healthy controls vs. all NSCLC	37.14 (25.89-49.52)	100 (91.59-100.00)	0.73
	All controls vs. >65 years old NSCLC	43.33 (25.46-62.57)	100 (94.87-100)	0.83
	All controls vs. <65 years old NSCLC	32.5 (18.57-49.13)	100 (94.87-100)	0.63
	All controls vs. early stages of NSCLC	33.33 (21.95-46.34)	100 (94.87-100)	0.71
	All controls vs. late stages of NSCLC	71.43 (29.04-96.33)	100 (94.87-100)	0.79
	All controls vs. LUAD	20.59 (8.70-37.90)	100 (94.87-100)	0.60
	All controls vs. LLCC	33.33 (0.84-90.57)	100 (94.87-100)	0.78
	All controls vs. LUSC	54.55 (36.35-71.89)	100 (94.87-100)	0.82
Cyfra 21-1	All controls vs. all NSCLC	61.43 (49.03-72.83)	80.00 (68.73-88.61)	0.76
	Benign controls vs. all NSCLC	61.43 (49.03-72.83)	89.29 (71.77- 97.73)	0.82
	Healthy controls vs. all NSCLC	61.43 (49.03-72.83)	73.81 (57.96-86.14)	0.72
	All controls vs. >65 years old NSCLC	66.67 (47.19-82.71)	80.00 (68.73-88.61)	0.79
	All controls vs. <65 years old NSCLC	57.5 (40.89-72.96)	80.00 (68.73-88.61)	0.74
	All controls vs. early stages of NSCLC	61.90 (48.80-73.85)	80.00 (68.73-88.61)	0.77
	All controls vs. late stages of NSCLC	57.14 (18.41-90.10)	80.00 (68.73-88.61)	0.68
	All controls vs. LUAD	35.29 (19.75-53.51)	80.00 (68.73-88.61)	0.60
	All controls vs. LLCC	100 (29.24-100.00)	80.00 (68.73-88.61)	0.98
	All controls vs. LUSC	84.85 (68.10-94.89)	80.00 (68.73-88.61)	0.91
HE4	All controls vs. all NSCLC	60.00 (47.59-71.53)	72.86 (60.90-82.80)	0.77
	Benign controls vs. all NSCLC	60.00 (47.59- 71.53)	64.29 (44.07- 81.36)	0.72
	Healthy controls vs. all NSCLC	60.00 (47.59- 71.53)	78.57 (63.19- 89.70)	0.81
	All controls vs. >65 years old NSCLC	73.33 (54.11-87.72)	72.86 (60.90-82.80)	0.85
	All controls vs. <65 years old NSCLC	50.00 (33.80-66.20)	72.86 (60.90-82.80)	0.71
	All controls vs. early stages of NSCLC	76.85 (67.75-84.43)	72.86 (60.90-82.80)	0.77
	All controls vs. late stages of NSCLC	57.14 (18.41-90.10)	72.86 (60.90-82.80)	0.76
	All controls vs. LUAD	35.29 (19.75-53.51)	72.86 (60.90-82.80)	0.66
	All controls vs. LLCC	100 (29.24-100.00)	72.86 (60.90-82.80)	0.99
	All controls vs. LUSC	81.82 (64.54-93.02)	72.86 (60.90-82.80)	0.88
SAA	All controls vs. all NSCLC	47.14 (35.09- 59.45)	92.86 (84.11- 97.64)	0.782
	Benign controls vs. all NSCLC	47.14 (35.09-59.45)	96.43 (81.65-99.91)	0.82
	Healthy controls vs. all NSCLC	47.14 (35.09- 59.45)	90.48 (77.38- 97.3)	0.75
	All controls vs. >65 years old NSCLC	53.33 (34.33-71.66)	92.86 (84.11- 97.64)	0.83
	All controls vs. <65 years old NSCLC	42.50 (27.04-59.11)	92.86 (84.11- 97.64)	0.74
	All controls vs. early stages of NSCLC	47.62 (34.88-60.59)	92.86 (84.11- 97.64)	0.77
	All controls vs. late stages of NSCLC	42.86 (9.90-81.59)	92.86 (84.11- 97.64)	0.85
	All controls vs. LUAD	41.18 (24.65-59.30)	92.86 (84.11- 97.64)	0.70
	All controls vs. LLCC	66.67 (9.43-99.16)	92.86 (84.11- 97.64)	0.83
	All controls vs. LUSC	54.55 (36.35-71.89)	92.86 (84.11- 97.64)	0.86

CEA: Carcinoma embryonic antigen; Cyfra 21-1: Cytokeratin 19-fragments; HE4: Human epididymis protein-4; SAA: Serum amyloid-A; NSCLC: Non-small-cell lung cancer; LUAD: Lung adenocarcinoma; LUSC: Lung squamous cell carcinoma; LLCC: Lung large cell carcinoma; CI: Confidence interval; AUC: Area under curve.

Table S6. Comparison of the effects of covariates on the diagnostic accuracy of biomarkers in the experimental validation step of the study.

Biomarker	Fixed feature	Variable feature	Compared variables	p-value
CEA	All NSCLC	Control group	All controls vs. Benign controls	0.69
			All controls vs. Healthy controls	0.77
	All controls	NSCLC subtype	LUAD vs. LLCC	0.016
			LUAD vs. LUSC	<0.01
			LUSC vs. LLCC	0.72
	All controls	Stage	Late vs. Early stages	0.52
	All controls	Age of NSCLC patients	>65 vs. <65 years old	0.008
Cyfra 21-1	All NSCLC	Control group	All controls vs. Benign controls	0.26
			All controls vs. Healthy controls	0.50
	All controls	NSCLC subtype	LUAD vs. LLCC	<0.001
			LUAD vs. LUSC	<0.001
			LUSC vs. LLCC	0.01
	All controls	Stage	Late vs. Early stages	0.48
	All controls	Age of NSCLC patients	>65 vs. <65 years old	0.04
HE4	All NSCLC	Control group	All controls vs. Benign controls	0.42
			All controls vs. Healthy controls	0.51
	All controls	NSCLC subtype	LUAD vs. LLCC	<0.001
			LUAD vs. LUSC	<0.001
			LUSC vs. LLCC	<0.001
	All controls	Stage	Late vs. Early stages	0.90
	All controls	Age of NSCLC patients	>65 vs. <65 years old	0.02
SAA	All NSCLC	Control group	All controls vs. Benign controls	0.41
			All controls vs. Healthy controls	0.60
	All controls	NSCLC subtype	LUAD vs. LLCC	0.04
			LUAD vs. LUSC	0.02
			LUSC vs. LLCC	0.83
	All controls	Stage	Late vs. Early stages	0.22
	All controls	Age of NSCLC patients	>65 vs. <65 years old	0.04

CEA: Carcinoma embryonic antigen; Cyfra 21-1: Cytokeratin 19-fragments; HE4: Human epididymis protein-4; SAA: Serum amyloid-A; NSCLC: Non-small-cell lung cancer; LUAD: Lung adenocarcinoma; LUSC: Lung squamous cell carcinoma; LLCC: Lung large cell carcinoma.

Table S7. Comparison of diagnostic accuracy of single and combined biomarkers in final step meta-analysis.

	**Relative Sensitivity**	**95% CI**	**Sensitivity p-value**	**Relative Specificity**	**95% CI**	**Specificity p-value**	**Global test comparison p-value**
Cyfra 21-1 vs CEA	1.22	(1.08-1.37)	0.001	0.99	(0.95-1.04)	0.77	0.003
HE4 vs CEA	1.44	(1.28-1.61)	<0.001	0.92	(0.87-0.99)	0.01	<0.001
SAA vs. CEA	1.41	(1.16-1.71)	0.007	0.94	(0.84-1.07)	0.27	0.02
CEA+Cyfra 21-1 21-1 vs CEA	1.28	(1.03-1.59)	0.05	0.91	(0.80-1.03)	0.07	0.08
CEA+HE4 vs CEA	1.20	(0.90-1.60)	0.28	0.97	(0.85-1.11)	0.67	0.55
HE4+Cyfra 21-1 21-1 vs CEA	1.57	(1.30-1.88)	0.002	0.90	(0.73-1.10)	0.17	0.007
CEA+Cyfra 21-1+HE4 vs CEA	1.53	(1.27-1.85)	<0.01	0.88	(0.71-1.08)	0.09	0.009
HE4 vs Cyfra 21-1	1.18	(1.08-1.29)	<0.01	0.93	(0.87-0.99)	0.01	<0.01
SAA vs Cyfra 21-1	1.15	(0.97-1.36)	0.15	0.95	(0.84-1.07)	0.31	0.31
CEA+Cyfra vs Cyfra 21-1	0.95	(0.79-1.15)	0.62	1.09	(0.97-1.24)	0.07	0.20
CEA+HE4 vs Cyfra 21-1	1.02	(0.78-1.33)	0.82	1.02	(0.90-1.16)	0.74	0.92
HE4+Cyfra 21-1 vs Cyfra 21-1	1.29	(1.10-1.51)	0.02	0.90	(0.74-1.10)	0.17	0.06
CEA+Cyfra 21-1+HE4 vs Cyfra 21-1	0.80	(0.68-0.94)	0.03	1.13	(0.93-1.38)	0.1	0.06
SAA vs HE4	0.98	(0.83-1.15)	0.79	1.03	(0.92-1.14)	0.65	0.90
CEA+Cyfra 21-1 vs HE4	1.12	(0.93-1.36)	0.21	1.01	(0.91-1.13)	0.83	0.39
CEA+HE4 vs HE4	1.20	(0.94-1.52)	0.10	0.95	(0.84-1.08)	0.47	0.26
HE4+Cyfra 21-1 vs HE4	1.09	(0.94-1.27)	0.31	0.97	(0.81-1.16)	0.71	0.60
CEA+Cyfra 21-1+HE4 vs HE4	0.94	(0.80-1.10)	0.46	1.05	(0.89-1.24)	0.55	0.73
CEA+Cyfra 21-1 vs SAA	1.11	(0.79-1.55)	0.56	1.04	(0.94-1.15)	0.46	0.54
CEA+HE4 vs SAA	1.18	(0.83-1.68)	0.34	0.98	(0.85-1.13)	0.78	0.62
HE4+Cyfra 21-1 vs SAA	0.91	(0.68-1.22)	0.55	1.07	(0.88-1.28)	0.50	0.77
CEA+Cyfra 21-1+HE4 vs SAA	0.92	(0.69-1.22)	0.58	1.07	(0.93-1.24)	0.34	0.64
HE4+Cyfra 21-1 vs CEA+Cyfra 21-1	1.22	(0.88-1.68)	0.26	0.96	(0.83-1.12)	0.63	0.52
CEA+HE4 vs CEA+Cyfra 21-1	0.93	(0.62-1.40)	0.72	1.06	(0.94-1.19)	0.38	0.64
CEA+Cyfra 21-1+HE4 vs CEA+Cyfra 21-1	1.20	(0.88-1.66)	0.29	0.98	(0.86-1.10)	0.69	0.54
HE4+Cyfra 21-1 vs CEA+HE4	1.31	(1.00-1.71)	0.08	0.92	(0.75-1.14)	0.46	0.21
CEA+HE4 vs CEA+Cyfra 21-1+HE4	0.79	(0.60-1.03)	0.8	1.10	(0.93-1.29)	0.27	0.22
HE4+Cyfra 21-1 vs CEA+Cyfra 21-1+HE4	1.02	(0.79-1.31)	0.91	1.01	(0.82-1.23)	0.97	0.99

CEA: Carcinoma embryonic antigen; Cyfra 21-1: Cytokeratin 19-fragments; HE4: Human epididymis protein-4; SAA: Serum amyloid-A; CI: Confidence interval.
